# Transcriptome Profiling of *Toxoplasma gondii*-Infected Human Cerebromicrovascular Endothelial Cell Response to Treatment with Monensin

**DOI:** 10.3390/microorganisms8060842

**Published:** 2020-06-04

**Authors:** Mohammad S. R. Harun, Mica Taylor, Xing-Quan Zhu, Hany M. Elsheikha

**Affiliations:** 1 Infectomics Cluster, Advanced Medical & Dental Institute, Universiti Sains Malaysia, Bertam, Kepala Batas, Pulau Pinang 13200, Malaysia; mosyamsulre@usm.my; 2 Faculty of Medicine and Health Sciences, School of Veterinary Medicine and Science, University of Nottingham, Sutton Bonington Campus, Loughborough LE12 5RD, UK; micataylor@hotmail.co.uk; 3 State Key Laboratory of Veterinary Etiological Biology, Key Laboratory of Veterinary Parasitology of Gansu Province, Lanzhou Veterinary Research Institute, Chinese Academy of Agricultural Sciences, Lanzhou 730046, China

**Keywords:** *Toxoplasma gondii*, monensin, TEER, gene expression, Wnt signaling

## Abstract

Central to the progression of cerebral toxoplasmosis is the interaction of *Toxoplasma gondii* with the blood-brain barrier (BBB) endothelial cells. In the present work, we tested the hypothesis that inhibition of Wnt pathway signalling by the monovalent ionophore monensin reduces the growth of *T. gondii* infecting human brain microvascular endothelial cells (hBMECs) or microglial cells. The anti-parasitic effect of monensin (a Wnt signalling inhibitor) on the in vitro growth of *T. gondii* tachyzoites was investigated using two methods (Sulforhodamine B staining and microscopic parasite counting). The monensin inhibited *T. gondii* growth (50% inhibitory concentration [IC_50_] = 0.61 μM) with a selective index = 8.48 when tested against hBMECs (50% cytotoxic concentration [CC_50_] = 5.17 μM). However, IC_50_ of monensin was 4.13 μM with a SI = 13.82 when tested against microglia cells (CC_50_ = 57.08 μM), suggesting less sensitivity of microglia cells to monensin treatment. The effect of *T. gondii* on the integrity of the BBB was assessed by the transendothelial electrical resistance (TEER) assay using an in vitro human BBB model. The results showed that *T. gondii* infection significantly decreased hBMECs’ TEER resistance, which was rescued when cells were treated with 0.1 µM monensin, probably due to the anti-parasitic activity of monensin. We also investigated the host-targeted effects of 0.1 µM monensin on global gene expression in hBMECs with or without *T. gondii* infection. Treatment of hBMECs with monensin did not significantly influence the expression of genes involved in the Wnt signalling pathway, suggesting that although inhibition of the Wnt signalling pathway did not play a significant role in *T. gondii* infection of hBMECs, monensin was still effective in limiting the growth of *T. gondii*. On the contrary, monensin treatment downregulated pathways related to steroids, cholesterol and protein biosynthesis and their transport between endoplasmic reticulum and Golgi apparatus, and deregulated pathways related to cell cycle and DNA synthesis and repair mechanisms. These results provide new insight into the host-modulatory effect of monensin during *T. gondii* infection, which merits further investigation.

## 1. Introduction

*Toxoplasma gondii* is an obligate intracellular apicomplexan protozoan parasite that is widely distributed throughout the world. The disease associated with *T. gondii* infection, toxoplasmosis, can lead to severe central nervous system (CNS) pathologies [1]. Although *T. gondii* infection is usually asymptomatic in healthy individuals, infection can be fatal in immunocompromised patients and in congenitally infected fetuses [2,3]. Given the pathological damage and neuroinflammation that can be caused by *T. gondii* infection of the brain [4,5], many epidemiological studies have shown an association between *T. gondii* infection and a number of neuropsychiatric conditions, such as schizophrenia and bipolar disorder [4,5,6]. Besides the lack of a vaccine, current treatment options for toxoplasmosis are limited and responses to commonly used medicines are often unsatisfactory [7]. There is a clear need for new and more potent anti-*T. gondii* therapeutics to reduce the clinical impact of toxoplasmosis. Ideal therapeutic agents should prevent parasite growth, without perturbing the proliferation or homeostasis of host cells. However, in general, although available therapeutic agents display potent anti-*T. gondii* properties, many are cytotoxic to mammalian cells [8].

The blood-brain barrier (BBB) is the initial site of interaction between neuro-pathogens such as *T. gondii* and the mammalian brain [9]. Breaching this protective biophysical barrier is a key mechanism by which *T. gondii* tachyzoites invade and damage the CNS [9]. During *T. gondii* infection, different signalling pathways control the expression of a wide range of genes that orchestrate molecular and cellular events to eliminate the invading parasite and regulate the associated inflammation [10]. One of these pathways is the Wnt pathway, which is considered an important regulatory axis in the immune system, where genes involved in this pathway were upregulated in *T. gondii*-infected mice [10]. Moreover, other intracellular pathogens have been shown to manipulate this pathway to facilitate their entry into the cell and establish infection, such as *Chlamydia trachomatis* [11] and the influenza virus [12].

Small molecule inhibitors of the Wnt pathway are becoming more prominent in the pharmaceutical industry [13], and are increasingly being used to target cancer cells and intracellular pathogens [14]. Natural polyether ionophores, such as monensin, have been substances of great interest, because of their antimicrobial activities [15]. Monensin has been extensively used in veterinary applications as a growth promoter [16] and to treat coccidiosis and cryptosporidiosis [15,17]. In addition, monensin was proposed as an anticancer agent [18,19]. Monensin, secreted by the bacteria *Streptomyces cinnamonensis*, has been shown to modulate Wnt signalling by antagonizing β-catenin and the lipoprotein receptor-related protein LRP [18]. Investigation into how manipulation of the Wnt pathway by monensin can influence the growth of *T. gondii* could be beneficial in the development of new anti-*T. gondii* therapeutics.

In this study, we investigated whether inhibition of the Wnt signalling pathway by monensin can reduce the growth of *T. gondii* infecting human brain microvascular endothelial cells (hBMECs) or microglial cells, and whether suppression of the growth of *T. gondii* within hBMECs using monensin can restore the impairment of the BBB integrity. We also performed systems-level transcriptional analysis of hBMECs infected by *T. gondii* in the presence or absence of monensin to obtain a comprehensive insight into how monensin treatment alters gene expression of hBMECs during *T. gondii* infection.

## 2. Materials and Methods

### 2.1. Chemicals

Monensin was purchased as a sodium salt powder from Alfa Aesar (Ward Hill, MA, USA) and 1.73 mg was dissolved in 250 μL of absolute methanol. The 10 mM of stock solution was diluted with complete culture medium to prepare five working concentrations 0.01 μM, 0.1 μM, 1 μM, 10 μM and 100 μM, which were stored at 4 °C until use. Sulforhodamine B (SRB) was purchased from Sigma-Aldrich (SIGMA-ALDRICH^®^/Merck KGaA, Darmstadt, Germany) and a solution of 0.4% (*w*/*v*) SRB dissolved in 1% acetic acid (Fisher Scientific, Leicestershire, UK) was prepared and stored protected from light at 4 °C.

### 2.2. Cell Lines

Primary human brain microvascular endothelial cells (hBMECs) kindly provided by Naveed Khan (American University of Sharjah, Sharjah, UAE) were maintained in modified Gibco^®^ Roswell Park Memorial Institute (RPMI) 1640 media (Thermo Fisher Scientific, Waltham, MA, USA) containing 20% Gibco^®^ Heat Inactivated Fetal Bovine Serum (HI-FBS), 1% Gibco^®^ non-essential amino-acids (100×), 1% Gibco^®^ sodium pyruvate (100 mM), 1% Gibco^®^ MEM vitamins (100×), 1% L-glutamine (200 mM), and 1% Gibco^®^ antibiotic-antimycotic solution 100× (10,000 units/mL of penicillin, 10,000 µg/mL of streptomycin, and 25 µg/mL of Amphotericin B). Human microglial cells (ATCC CRL-3304) originally obtained from the American Type Culture Collection were grown in modified Gibco^®^ Dulbecco’s Modified Eagle Medium (DMEM; Thermo Fisher Scientific, Waltham, MA, USA) containing 5% Gibco^®^ HI-FBS and 1% Gibco^®^ antibiotic-antimycotic solution 100X. Both cell lines were passaged twice a week and grown in T75 (75 cm^2^) NUNC™ tissue culture flasks (Fisher Scientific, Leicestershire, UK) for two weeks before being used in the experiments. All cultures were maintained in a cell culture incubator at 37 °C with 5% atmospheric CO_2_.

### 2.3. Parasite Strain and Culture Conditions

Tachyzoites of the *T. gondii* RH strain were cultured in NUNC™ T75 tissue culture flasks containing hBMECs using the same cell culture conditions described above. The tachyzoites were harvested from their feeder cell cultures when 70–80% of the hBMECs were lysed in about three days. Then, they were separated from host cell debris via centrifugation at 500 *g* for 5 min. Based on the cell line used in the subsequent experiments, the number of tachyzoites was adjusted with the respective medium (RPMI for hBMECs or DMEM for microglial cells) to achieve a multiplicity of infection (MOI) of 5 (5 tachyzoites: 1 cell) for each cell line.

### 2.4. Efficacy of Monensin on the In Vitro Growth of T. gondii

We used parasite counting assay in 24-well plates and the sulforhodamine B (SRB) assay in 96-well plates to investigate the effects of inhibition of the Wnt signalling pathway using monensin on the intracellular growth of *T. gondii*.

#### 2.4.1. Parasite Counting Assay

Microglial and hBMEC cultures were seeded on Nunclon™ Delta Surface 24-well culture plates (Thermo Fisher Scientific, Waltham, MA, USA) at seeding densities of 5 × 10^4^ cells and 10^5^ cells per well, respectively. Cultures of both cell types were infected with *T. gondii* tachyzoites at a MOI of 5. Approximately 3 h post-infection, the culture medium in each well was discarded and replaced with 1 mL of fresh medium supplemented with one of the five working concentrations of monensin (0.01 μM, 0.1 μM, 1 μM, 10 μM and 100 μM). Growth of cell cultures was monitored daily by microscopic observation using a Zeiss Axiovert 25 inverted microscope (Leica Microsystems, Milton Keynes, UK) with a 10X objective lens. When the parasites had completed a few cycles of development and began to exit the cells in large numbers at ~3 days after infection of the untreated cells, the absolute number of tachyzoites in each well of treated and untreated cultures was counted. To achieve this, the remaining medium in the wells was topped up with phosphate buffered saline (PBS) with pipetting to produce a homogenous parasite suspension. A small amount (50 μL) of the suspension from each well was used to count tachyzoites using a standard hemocytometer counting slide.

#### 2.4.2. Colorimetric SRB Assay

The SRB assay involves the measurement of cellular protein content using a plate reader to quantify the absorbance after cells have been fixed and stained with SRB dye. The SRB absorbance values represent the spectrophotometric quantification of protein concentration of the cells, which is directly proportional to the number of cells (i.e. increased absorbance correlates with increased protein content, which reflects an increase in the cells’ number). Hence, SRB absorbance values can be used as an indicator of host cell proliferation rates in *T. gondii*-infected versus mock-infected (control) cultures to determine the level of *T. gondii* growth indirectly. Based on this rationale, we can expect infected cells to produce less absorbance due to the disruption of cell proliferation caused by the damaging effects of the parasite growth on the host cell viability. Here, a microplate-based SRB staining assay was used as a simple, cost-effective, convenient approach to determine the inhibitory effect of monensin on *T. gondii* growth in vitro. Briefly, cells were cultured in Corning^®^ Costar^®^ 96-well plates (Sigma-Aldrich/Merck KGaA, Darmstadt, Germany). Each well was seeded with 5 × 10^3^ microglial cells or 10^4^ hBMECs in 100 µL of the respective medium and then grown to confluency (~1 day). Tachyzoites used to infect each well at a MOI of 5 were able to invade the host cells within 3 h. Then, the medium was discarded to remove any extracellular tachyzoites and 100 µL of media containing one of the five concentrations of monensin (0.01 μM, 0.1 μM, 1 μM, 10 μM, and 100 µM) was added to each well. Wells that received medium without monensin (untreated) served as controls. After five days of incubation, the SRB assay was performed according to Vichai and Kirtikara [20]. Ice-cold 100 µM of 10% trichloroacetic acid (TCA) solution (Fisher Scientific, Leicestershire, UK) was added to each well. The plates were kept at 4 °C for 1 h. Afterwards, the solution was discarded from the wells followed by thorough washing with distilled water. Next, 25 µL of SRB dye was added to each well and incubated for 15 min at ambient temperature. The plates were wrapped with foil to protect the dye from the light. Then, the wells were washed with 1% acetic acid to remove any unattached stain, followed by addition of 100 µL of 10 mM Tris base solution (Sigma-Aldrich/Merck KGaA, Darmstadt, Germany) to each well. Finally, the plates were gently agitated on a plate shaker at 35 rotations/min for 5 min to ensure a homogeneous solution. The absorbance was measured at 492 nm using a plate reader (LT-4000 Microplate Reader, LabTech International Ltd., Heathfield, UK). 

### 2.5. Effect of Monensin on BBB Integrity

The hBMECs are fundamental constituents of the BBB and play important roles in the maintenance of barrier integrity. To determine if and to what extent integrity of hBMECs is affected by exposure to 0.1 µM monensin in the presence or absence of *T. gondii* infection, transendothelial electrical resistance (TEER) was measured. Briefly, 0.5 µM PET-inserts (24-well Millicell^®^ Cell Culture Inserts, Millipore, Watford, UK) were fitted to wells of the 24-well culture plates. Then, 10^4^ cells in 100 µL of RPMI medium were added to each insert and 100 μL of the culture medium was added to the bottom of the well. The plate was incubated for 1 h; then the medium was discarded and replaced with 1.3 mL fresh media in the bottom of each well. Also, 300 µL fresh media was added onto the cell monolayer inside the inserts. Three days after seeding the cells, the media in the inserts and wells were discarded and replaced with fresh media. Six days after seeding, one of the plates was infected with *T. gondii* by removing old media and adding tachyzoites at a MOI of 5, in fresh medium, to the inserts. The plate was incubated for 1 h before addition of monensin-supplemented RPMI or fresh RPMI (control) to hBMEC monolayer growing on the apical (top) side of insert. TEER was measured in each insert daily for 4 successive days. TEER was measured using an Evom voltohmmeter with the Endohm 12 and STX2 electrodes (World Precision Instruments, Inc., Sarasota, FL, USA). The electrodes were placed into the insert membrane and bottom of each well. TEER values were measured three times from each well, while changing the position of the electrodes slightly each time in order to obtain the overall average of the insert resistance. A background resistance was established by placing the electrodes into inserts with a blank medium only, without cells. This value was used to calculate the TEER value of each well using the equation (TEER (cells) = (TEER(total) − TEER(blank)) culture area). The culture area of the insert was 0.33 cm^2^. The TEER value was expected to increase with increased resistance of the hBMEC monolayer.

### 2.6. Microarray Analysis

The aim of this experiment was to identify the effect of 0.1 µM monensin on the overall expression of host cell genes, and in particular on the expression of genes involved in the Wnt signalling pathway. We used cDNA microarrays to detect changes in mRNA expression in four experimental groups. These included: uninfected, un-treated hBMECs (Control); hBMECs treated with 0.1 µM monensin for 24 h (Monensin_only); *T. gondii* infected-hBMECs for 24 h (TG_only); and *T. gondii*-infected hBMECs treated with 0.1µM monensin after 3 h of infection and incubated for further 24 h (TG_Monensin).

Total RNA was isolated using RNeasy Mini Kit (Qiagen, Manchester, UK) and eluted with nuclease-free water. The extracted RNA was stored at −80 °C for one week prior to microarray analysis. All subsequent sample handling, labelling, and GeneChip (GeneChip Human Gene 1.0 ST arrays, Affymetrix, Santa Clara, CA, USA) processing were performed at the transcriptomics service at Nottingham Arabidopsis Stock Centre (NASC). The RNA concentration and quality were assessed using the Agilent 2100 Bioanalyzer (Agilent Technologies Inc., Palo Alto, CA, USA) and the RNA 6000 Nano kit (Caliper Life Sciences, Mountain View, CA, USA), respectively, prior to the microarray analysis. Only RNA samples with a minimum RNA concentration of 100 ng/µL and RNA integrity number (RIN) value > 8 were used for microarray analysis.

Single-stranded complimentary DNA was prepared from 200 ng of total RNA as per the Ambion^®^ WT Expression Kit’s instructions (Thermo Fisher Scientific, Paisley, UK) for Affymetrix GeneChip Whole Transcript WT Expression Arrays (Affymetrix, Wycombe, UK). Total RNA was first converted to cDNA, followed by transcription to make cDNA. Single-stranded cDNA was synthesized, end labelled, and hybridized for 16 h at 45 °C to Human Gene 1.0 ST arrays (Affymetrix, Wycombe, UK). All liquid handling steps were performed by a GeneChip Fluidics Station 450 and GeneChips were scanned with the GeneChip Scanner 3000 7G (Affymetrix, Wycombe, UK) using Command Console v3.2.4. The microarray data have been deposited in the ArrayExpress database at EMBL-EBI (Available online: www.ebi.ac.uk/arrayexpress) under accession number E-MTAB-8817.

The R package software (R Studio Inc., Boston, MA, USA) was used to process the data. Briefly, a data file, called ‘pdatamon’ was created to link each microarray data file with ‘.ga.cel’ ending to one of the four experimental groups (i.e. Control, Monensin_only, TG_only, TG_Monensin). The libraries of modules (oligo, gplots, RColorBrewer, pvclust, colourspace, limma, gene filter, hugene21sttranscriptcluster.db) needed for quality control, differential gene expression and transcript cluster ID annotation were downloaded and uploaded into the workspace. Next, Affymetrix microarray data files were uploaded together with ‘pdatamon’ file. Quality control analysis and normalization were performed using the robust multi-array average (RMA). RMA normalization is sensitive for low expression values, and provides consistent fold-change estimates, and excellent background adjustment compared to MAS 5.0 and dChip normalization methods [21,22]. Only genes exhibiting a fold-change FC ≤ −1 (downregulated) or FC ≥ 1 (upregulated) relative to control and an adjusted *p*-value < 0.05 were considered as differentially expressed genes (DEGs). The ranked list of DEGs were included in subsequent analyses. Comparison was made between control (untreated + uninfected) hBMECs and each of the other three groups, including hBMECs treated with monensin (Monensin_only), *T. gondii*-infected hBMECs treated with monensin (TG_Monensin), and hBMECs infected with *T. gondii* only (TG_only).

### 2.7. Gene Ontology (GO) Analysis

Functional annotations of the valid DEGs and statistical overrepresentation of the GO terms in the categories of biological processes, molecular functions, and cell components [23], were retrieved in accordance with instructions [24,25], using the PANTHER online database (Available online: www.pantherdb.org). The PANTHER library of annotated genes (*Homo sapiens*) was used as reference list for calculating statistical overrepresentation with a false discovery rate (FDR) < 0.05 considered significantly enriched.

### 2.8. Pathway Enrichment Analysis

The DEGs genes were functionally annotated and positioned within the respective biological pathways using the Reactome pathway database (Available online: https://reactome.org) and the Reactome Analysis Tool version v65 (Available online: https://reactome.org/PathwayBrowser/#TOOL=AT). Significance was estimated using hypergeometric testing and FDR was controlled for using the Benjamini–Hochberg procedure. Pathways with FDR < 0.01 were considered significantly enriched.

### 2.9. Statistical Analysis

Statistical analysis was performed using a Student’s *t*-test (two independent groups), one-way ANOVA (multiple groups, one independent factor) or two-way ANOVA (multiple groups, two independent factors) followed by a Bonferroni–Holm post hoc test using GraphPad Prism 7 software for Windows (GraphPad Software, San Diego, CA, USA). The mean of at least three independent experiments is presented, with error bars in the graphs showing the standard error of the mean (SEM). Differences were considered statistically significant at a *p* value of < 0.05. The level of significance was reported as: * *p* < 0.05, ** *p* < 0.01, *** *p* < 0.001, and **** *p* < 0.0001. The drug concentration that caused 50% inhibition of host cell growth was expressed as 50% cytotoxic concentration (CC_50_). The CC_50_ of monensin on microglia and hBMECs were calculated by plotting dose-response curves followed by performing simple linear regression analysis using Graph Pad Prism 7 software. The calculation of the half-maximal inhibitory concentration (IC_50_), which is the concentration of monensin that caused a 50% decrease of *T. gondii* growth compared to the control in both cell lines, were calculated using Graph Pad Prism 7’s nonlinear regression (curve fit) built-in analysis tool of dose-response inhibition (three parameters). The selectivity index (SI), which represents the ratio of the CC_50_ for host cells to the IC_50_ for *T. gondii*, was calculated by comparing the cytotoxicity of monensin for host cells to that of *T. gondii*.

## 3. Results

### 3.1. Antiparasitic Effect of Monensin

**Figure 1:**
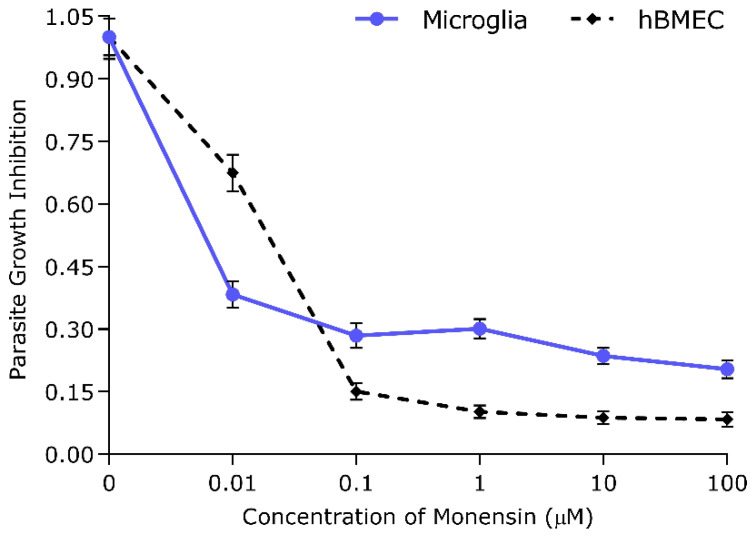
*Toxoplasma gondii* tachyzoite growth inhibition in microglia and human brain microvascular endothelial cell (hBMEC) cultures was dependent on monensin concentration. The parasite growth was inhibited (compared to parasite growth in untreated cells) as the concentration of monensin increased, but at 0.1 µM the inhibitory effect reached a plateau. Data represent mean values (± SEM) of triplicate from two independent experiments for each cell line.

The tachyzoite counts were carried out to measure the effect of monensin on the in vitro growth of *T. gondii*. The results showed a significant decrease (*p* < 0.01; ANOVA) in the number of counted tachyzoites in a dose-dependent manner, and >90% and 70% inhibition of *T. gondii* was achieved at 1 µM monensin concentration in hBMECs and microglial cells, respectively. The inhibitory effect of monensin on parasite growth was relatively greater in infected hBMEC cultures from 0.1 µM to 100 µM concentration. However, 0.1 µM was considered the minimal concentration to cause significant antiparasitic effects because no significant reduction in the tachyzoite counts was caused by higher concentrations and the reduction was not significantly different between hBMECs and microglia cells (Figure 1).

**Figure 2:**
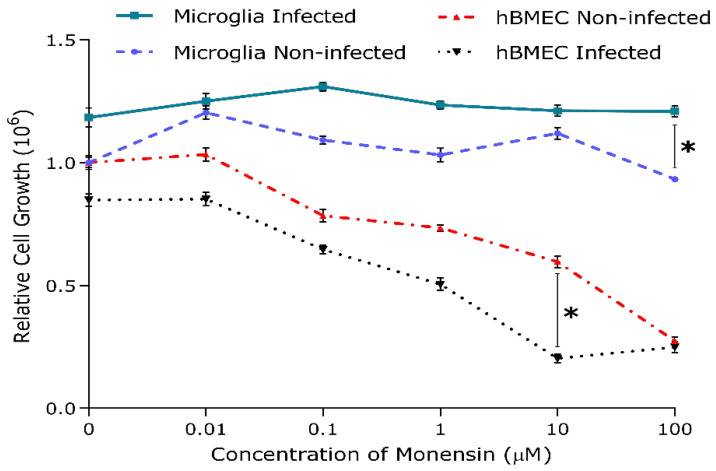
Microglia and hBMEC cultures showed different sensitivity to monensin. Relative growth of each cell line was assessed by the sulforhodamine B (SRB) assay. The relative absorbance values of infected and treated cells were compared to non-infected and treated (control) cells. A significant decrease in cell number was observed in infected and uninfected hBMECs with increasing monensin concentration compared to microglia cultures. Data represent mean values (± SEM) of five replicates from three independent experiments for each cell line. Multiple Student *t*-tests; *, *p* < 0.05.

It was important to test the effects of monensin on hBMECs and microglia in the absence of *T. gondii* infection. The SRB-based results showed lack of significant difference in cell density levels between infected and non-infected cultures of both hBMECs and microglia at low levels of monensin, suggesting that the number of treated + infected cells did not significantly decrease more than the control cells, probably due to inhibition of *T. gondii* infection by monensin (Figure 2). We performed *t*-tests to draw comparisons on the effect of each concentration of monensin between infected and non-infected cultures of each cell line. None of the data showed any significant differences except the 10 µM treated-hBMEC cultures and 100 µM treated-microglia cultures. The density of both infected and non-infected hBMEC cultures showed significant decrease (*p* < 0.05) within the entire range of the tested concentrations (0.01 µM to 100 µM). However, when the same SRB assay was performed on microglia culture (infected and non-infected), there was no significance reduction in cell density.

It is worthwhile to highlight that the SRB approach does not distinguish between anti-*T. gondii* effect and host cell toxicity characteristics, since death of host cells carrying the parasite could be related to cell toxicity caused by monensin as well as, or in lieu of, any anti-*T. gondii* effect. Therefore, it is possible that the anti-*T. gondii* effect of monensin in infected hBMECs may be confounded by the toxic effect of monensin on these cells. The 50% inhibitory concentration (IC_50_) for *T. gondii* tachyzoites (0.61 μM) and half-maximal cytotoxic concentrations (CC_50_) for hBMECs was 5.17 μM. The selective index (SI) was calculated as the CC_50_/IC_50_ of monensin and was found to be 8.48. However, the observed effects of monensin seem to be specific to host cell sensitivity to monensin and independent of the direct anti-*T. gondii* effect, since treatment with monensin did not induce the same cytotoxic effect in microglia cultures, where IC_50_ of monensin was 4.13 μM with a SI = 13.82 when tested against microglia (CC_50_ = 57.08 μM). These results show that although monensin reduced the growth rates of *T. gondii* tachyzoites in both hBMECs and microglia cells, the SI was higher in microglia cells compared to hBMECs, suggesting less sensitivity of microglia cells to monensin treatment.

### 3.2. Assessment of hBMEC Monolayer Integrity

**Figure 3:**
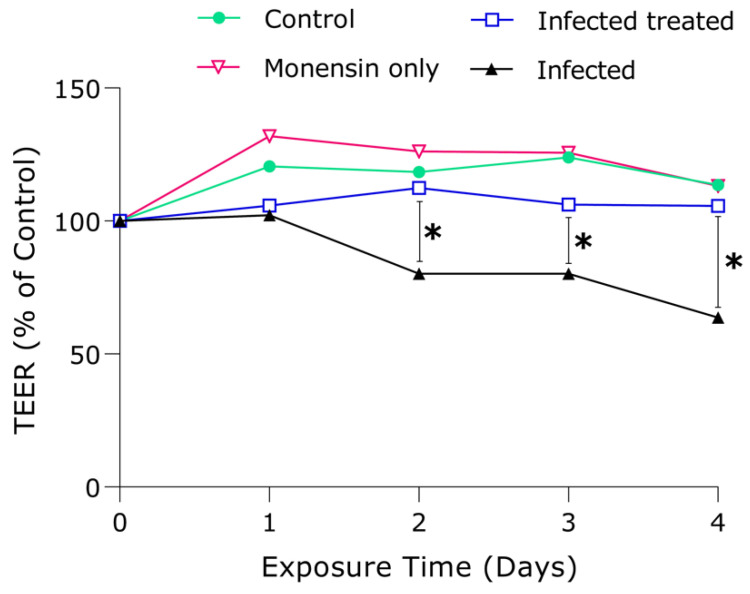
The effect of 0.1 µM monensin on the transendothelial electrical resistance (TEER) of hBMEC monolayer. Cells grown on the membrane of transwell inserts, a simplified blood-brain barrier model, were treated with 0.1 µM monensin or medium only (control) in the presence or absence of *T. gondii* infection. Infected and monensin-treated monolayer maintained their TEER value over four days, but, in non-treated and infected monolayer, a significant reduction in TEER values was observed (Multiple Student *t*-tests; *, *p* < 0.05). The data shown represent the means of six replicates from two separate experiments and the error bars indicate the S.E.M.

We investigated how hBMEC integrity was affected by *T. gondii* infection and/or modulated by Wnt inhibition via exposure to 0.1 µM monensin. The effect of monensin on the TEER of infected and uninfected hBMECs was monitored over 4 days. At one day post infection, there was no significant (*p* ≥ 0.05; *t*-tests) difference between *T. gondii*-infected cells treated with 0.1 µM monensin compared to infected + untreated, probably because it takes about a day for infection to establish. However, from day 2 after infection there was a significant decrease in hBMECs’ TEER between 0.1 µM monensin-treated and untreated infected cells. Interestingly, infected and treated cells did not show any significant difference throughout the entire experiment, suggesting that cell integrity was maintained by monensin treatment, possibly via inhibiting the damage caused by the parasite growth (Figure 3). On the contrary, the infected and untreated cells showed a significant reduction in TEER readings compared to the infected and treated cells (*p* < 0.05), suggesting that *T. gondii* infection significantly compromised host cell integrity. There was a 38.2% decrease in the resistance of infected + untreated cells compared to infected + treated cells relative to the control cells. Interestingly, hBMECs’ resistance did not change significantly from day to day and cell integrity was generally stable over the time period for control, infected + treated, and 0.1 µM monensin only groups. These findings are in line with the results of the anti-*T. gondii* effect of monensin (Figure 1 and Figure 2), suggesting that monensin had offset the cellular damage through inhibition of the parasite growth within the hBMECs and, hence, enhanced the recovery of cell integrity, leading to better endothelial resistance than infected + untreated cells.

### 3.3. Microarray Quality Control Analysis

The transcriptome analysis included four experimental groups: (i) uninfected and untreated hBMECs (Control—C2YA; C2YB; C2YC); (ii) uninfected hBMECs treated for 24 h with 0.1 μM monensin (Monensin_only—0.1μM_A; 0.1μM_B; 0.1μM_C); (iii) hBMECs infected with *T. gondii* only (TG_only—Inf 24 A; Inf 24 B; Inf 24 C); and (iv) *T. gondii*-infected hBMECs treated for 24 h with 0.1 μM monensin after 3 h of infection (TG_Monensin—TG + 0.1μMA; TG + 0.1μMB; TG + 0.1μMC). The 0.1 μM concentration was selected for the array experiment because preliminary data revealed that this concentration was just sufficient to trigger the cell’s transcriptional response (unpublished data) and also to avoid the growth inhibition of hBMECs that would have occurred at higher concentrations. The overall distribution between microarray files of the examined samples indicated that normalization was required before the samples are analyzed. As shown in the pre-normalization box plot (Figure S1A), the distributions of all sample median expression values were not uniform before normalization, which might lead to under- or over-estimated expression values if samples were compared. Following normalization, the median expression values of all samples became more uniform (Figure S1B).

### 3.4. Correlation Between Samples

**Figure 4:**
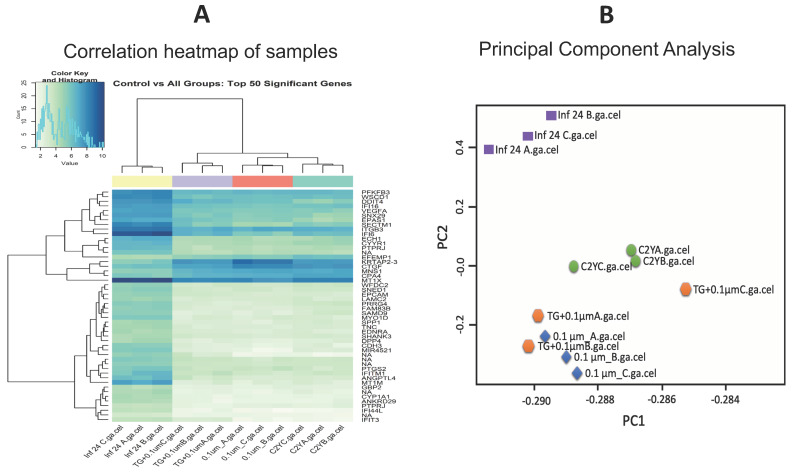
(**A**) A heatmap showing the correlation between all 12 microarray transcriptome expression profiles based on the top 50 most significantly expressed genes. Shades of light green and dark blue represent low and high correlation, respectively. Based on the dendrogram, four groups of the 12 sample expression profiles were clustered into four distinct clusters, where three samples within each group had similar expression profiles. (**B**) Principal component analysis (PCA) plot also shows that samples within each group were similar to each other, with the only exception being the dataset of TG_Monensin group samples.

**Figure 5:**
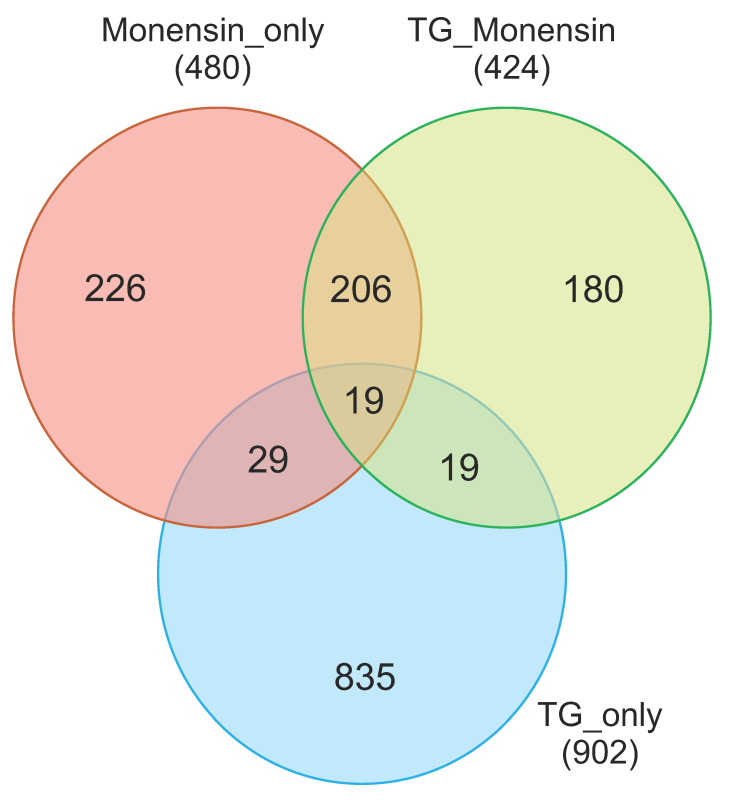
Three-way Venn diagram comparing expression profiles of Control vs. TG_only, Control vs. Monensin_only and Control vs. TG_Monensin. Numbers in the overlapped areas (i.e. at the intersection of the circles) represent DEGs shared between groups. The shaded area in the middle represents 19 DEGs shared between the three groups (Table S1).

Hierarchical clustering was performed to show the level of correlation between the transcriptional profiles of all 12 samples (based on the top 50 most significantly expressed genes) and identified four distinct subgroups (Figure 4A). Based on the dendrogram, four experimental groups, representing 12 expression profiles, were segregated into four clusters. These four clusters corresponded to four groups (TG_only, TG_Monensin, Monensin_only, Control_only) where three replicates within each group had similar expression patterns. As shown in the dendrogram, the expression profile of TG_only group was different from the expression profiles of the other three groups, namely Control_only, Monensin_only and TG_Monensin. Principal component analysis (PCA) also showed similar clustering of samples into four distinct groups, where samples within each group were clustered together. However, despite the high degree of concordance between the replicates per group, the TG + 0.1µM_C sample did not fully cluster with the other two replicates of the TG_Monensin group, which might be attributed to sample to sample variation within the group (Figure 4B). Moreover, TG_Monensin group and Monensin_only group samples clustered together and their gene expression profiles were more similar than those of the TG_only group samples. The overall expression values between the three experimental groups compared to the Control_only group is displayed as a Venn diagram (Figure 5). This diagram shows the shared and unique DEGs between the three comparison groups. Table S1 lists 19 DEGs that are shared by all three comparison groups. The TG_only had more total DEGs compared to the other two groups (Monensin_only and TG_Monensin).

### 3.5. Expression Patterns of Altered Genes

Here, the transcriptional response of hBMECs treated with monensin in the presence or absence of *T. gondii* infection was investigated in order to obtain more insight into the mechanism of action of monensin against *T. gondii*.

#### 3.5.1. Control vs. Monensin_only

**Figure 6:**
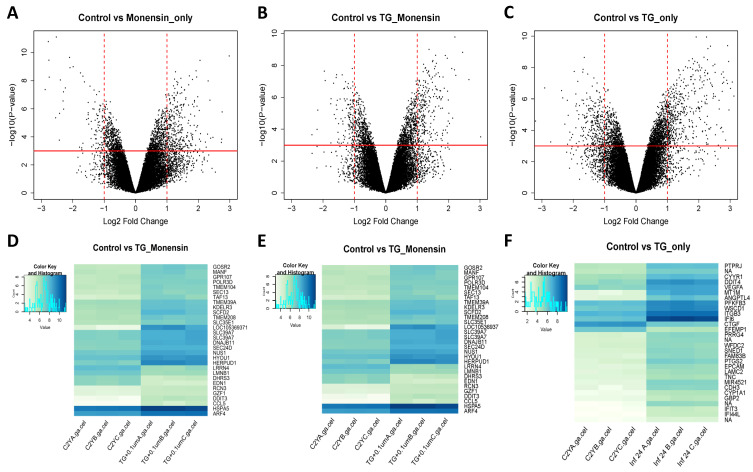
(**A**–**C**) Volcano plots showing the expression profile of hBMECs treated with 0.1 μM monensin (Monensin_only), hBMECs treated with 0.1 μM monensin + infected by *T. gondii* (TG_Monensin), and hBMECs infected by *T. gondii* (TG_only), compared to control hBMECs. The red horizontal line represents a cut-off value of *p* < 0.05 and the vertical dash red lines represent the log_2_ FC cut-off value of ≤ −1 (downregulated) or ≥ 1 (upregulated). Generally, more upregulated genes passed both cut-off values than down-regulated genes. (**D**–**F**) Correlation heatmaps of the top 30 most significant DEGs following treatment with monensin, following treatment + infection, and following infection, compared to control. The rows represent DEGs and the columns represent the 12 examined samples. The dendrogram shows a high intra-sample correlation between each group for all groups. Genes labelled as NA (not available) denote unpublished genes or uncharacterized non-coding RNA genes without annotation.

Treatment of hBMECs with 0.1 μM monensin induced more upregulation of genes than downregulation (Figure 6A). A total of 480 DEGs were identified; 368 were upregulated and 112 were downregulated. Among the 480 DEGs, 244 were not annotated (NA) and 236 were annotated. Out of the annotated genes, 171 were upregulated and 65 were downregulated. The top 30 most significant DEGs induced by monensin treatment and their name, gene symbol, gene description, and log_2_ FC values are listed in Table S2. The most significantly annotated gene was ‘DNA damage inducible transcript 3′ (*DDIT3*) with a log_2_ FC of 2.984. In addition, the top 30 upregulated and downregulated genes are listed in Tables S3 and S4, respectively. Among these, the most highly upregulated gene was also *DDIT3*, whereas the most highly downregulated gene with annotation was ‘small nucleolar RNA, C/D box 116-24′ (*SNORD116-24*) with a log_2_ FC of −2.543. The heatmap shows a correlation between samples based on the top 30 most significant DEGs after 24 h treatment with 0.1 μM monensin (Figure 6D).

#### 3.5.2. Control vs. TG_Monensin

The overall expression profile of hBMECs infected with *T. gondii* and treated with 0.1 μM monensin (TG_Monensin) for 24 h is shown (Figure 6B). Overall, there were more upregulated genes than downregulated genes. A total of 424 DEGs passed the selection criteria, of these 134 were upregulated and 290 were downregulated. Among the 424 DEGs, 114 were without annotation (NA) while 310 were annotated. Focusing on the annotated genes, 201 of them were upregulated, but 109 were downregulated. The top 30 most significant DEGs in hBMECs infected with *T. gondii* and treated with 0.1 μM monensin (TG_Monensin) are listed in Table S5. Among these, the most highly significant upregulated genes with annotation were *DDIT3* with a log_2_ FC of 2.446 and ‘homocysteine inducible ER protein with ubiquitin-like domain 1′ (*HERPUD1*) with a log_2_ FC of 2.199. The top 30 upregulated and downregulated genes are listed in Tables S6 and S7, respectively. The most upregulated gene was ‘uncharacterized LOC105369371′ (*LOC105369371*) with a log_2_ FC of 5.135 and the most downregulated gene was ‘endothelin 1′ (*EDN1*) with a log_2_ FC of −1.960. Figure 6E depicts the heatmap showing a correlation between samples based on to the top 30 most significant DEGs in hBMECs infected with *T. gondii* and treated with 0.1 μM monensin (TG_Monensin).

#### 3.5.3. Control vs. TG_only

The overall expression profile of hBMECs infected with *T. gondii* (TG_only) for 24 h showed that more genes were upregulated than downregulated (Figure 6C). The total number of genes that passed selection criteria was 902 genes, with 353 of them were upregulated while 549 were downregulated. Among the 902 DEGs, 454 were without annotation (NA), while the remaining 448 were annotated. For the annotated genes, 345 of them were upregulated, whereas 103 were downregulated. The top 30 most significant DEGs from TG_only samples are listed in Table S8. Among these, the most highly significant upregulated gene with annotation was ‘epithelial cell adhesion molecule’ (*EPCAM*) with a log_2_ FC of 2.249. The top 30 upregulated and downregulated genes are listed in Tables S9 and S10, respectively. Among these, the most highly upregulated gene with annotation was ‘metallothionein 1M’ (*MT1M*) with a log_2_ FC of 4.414 and the most highly downregulated gene with annotation was ‘microRNA 29a’ (*MIR29A*) with a log_2_ FC of −3.201. Figure 6F depicts the heatmap showing a correlation between samples based on the top 30 most significant DEGs from 24 h *T. gondii* infection.

### 3.6. Gene Ontology (GO) and Pathway Analysis

Here, the transcriptional changes in hBMECs in response to treatment with 0.1 μM monensin (Monensin_only), *T. gondii* infection (TG_only), and *T. gondii* infection plus treatment with 0.1 μM monensin (TG_Monensin) were investigated. For this analysis, only annotated genes were considered. Out of 112 downregulated genes, only 65 (58.03%) were annotated, whereas 171 out of 368 (46.47%) upregulated genes were annotated in the Monensin_only group. Regarding Control vs. TG_Monensin, 109 out of 134 (81.34%) downregulated genes were annotated, and 201 out of 290 (69.31%) up-regulated genes were annotated. For Control vs. TG_only, 103 out of 353 (29.18%) downregulated genes were annotated, but 345 out of 549 (62.84%) upregulated genes were annotated.

#### 3.6.1. Control vs. Monensin_only

**Figure 7:**
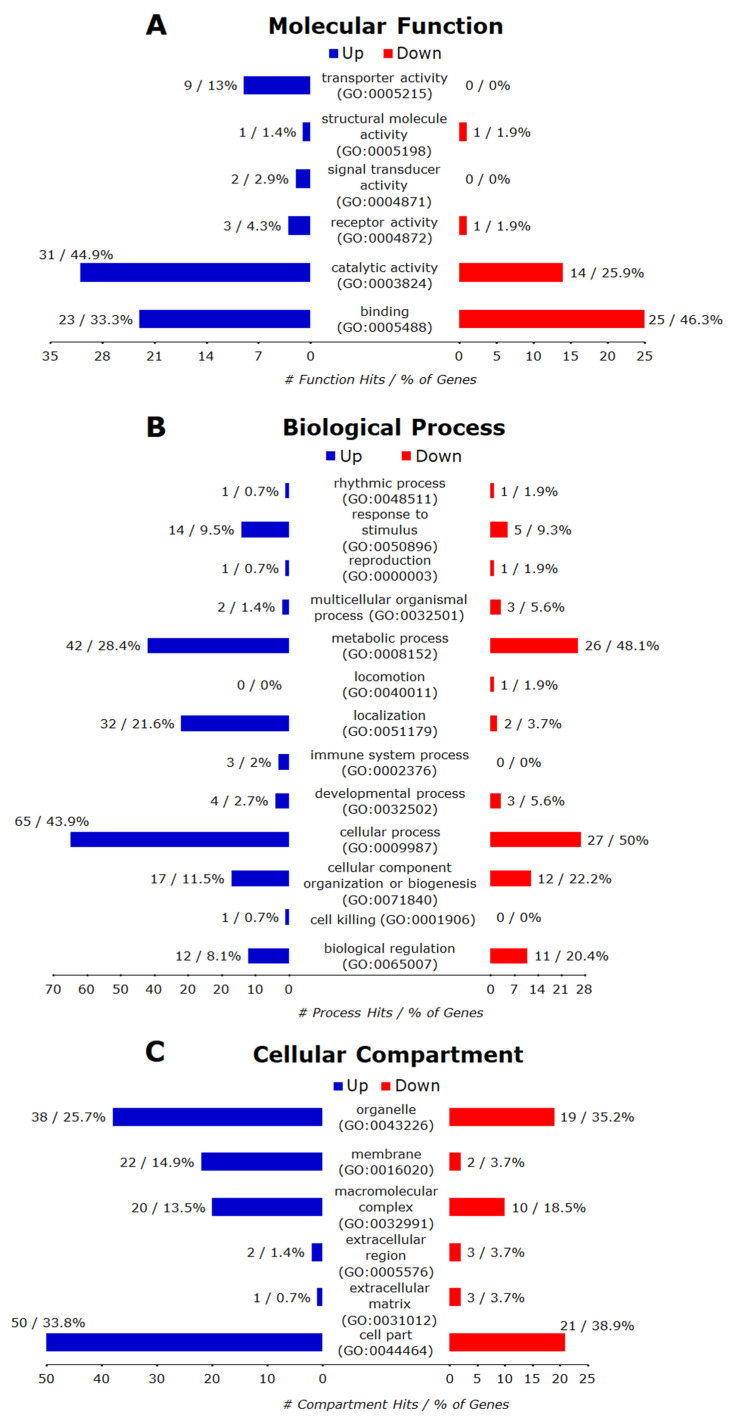
PANTHER GO-Slim gene ontology hits for significant DEGs due to treatment with 0.1 µM monensin for 24 h (Monensin_only). (**A**) The down-regulated genes matched four MF terms and the up-regulated genes matched six MF terms. The PANTHER Overrepresentation Test (FDR < 0.05) identified five and one overrepresented MF terms for down- and up-regulated genes, respectively. (**B**) The down-regulated genes matched 11 BPs and the up-regulated genes matched 12 BPs. The PANTHER Overrepresentation Test (FDR < 0.05) identified 11 and 10 overrepresented BPs for down- and up-regulated genes, respectively. (**C**) The down-regulated genes matched six CCs while up-regulated genes matched six CCs. The PANTHER Overrepresentation Test (FDR < 0.05) identified one and six overrepresented CC terms for down- and up-regulated genes, respectively.

Of the 65 annotated downregulated genes, 54 were recognized by PANTHER database and the GO terms belonging to the categories of biological process (BP), molecular function (MF), cellular compartment (CC) are presented in Figure 7A–C, respectively. Overall, 54 genes matched 11 GO terms of BP, including cellular process, metabolic process, cellular component organization or biogenesis, biological regulation, response to stimulus, developmental process, multicellular organismal process, localization, reproduction, rhythmic process, and locomotion. Using PANTHER Overrepresentation Test with the default setting (Fisher’s Exact with FDR multiple test correction) for PANTHER GO-Slim Biological Process, 11 out of 92 BPs were remarkably enriched (FDR < 0.05). These included DNA metabolic process, cell cycle, regulation of cell cycle, mitosis, nucleobase-containing compound metabolic process, DNA replication, chromosome segregation, organelle organization, cell proliferation, chromatin organization, and metabolic process. The downregulated genes also matched to four MF terms (binding, catalytic activity, receptor activity and structural molecule activity). The PANTHER Overrepresentation Test identified DNA helicase activity, helicase activity, kinase regulator activity, nucleic acid binding and binding as overrepresented MFs. In the CC ontology, downregulated genes matched six CC terms (cell part, organelle, macromolecular complex, membrane, extracellular matrix and extracellular region). The PANTHER Overrepresentation Test identified the nucleus as the only overrepresented CC.

Of the 171 annotated upregulated genes, 148 were recognized by the PANTHER database and the highly enriched terms for three GO categories (BP, MF, CC) are presented in Figure 7A–C, respectively. Overall, these genes matched to 12 BP 12 terms (cellular process, metabolic process, localization, cellular component organization or biogenesis, response to stimulus, biological regulation, developmental process, immune system process, multicellular organismal process, reproduction, rhythmic process, cell killing), six MFs terms (catalytic activity, binding, transporter activity, receptor activity, signal transducer activity, structural molecule activity) and six CC terms (cell part, organelle, membrane, macromolecular complex, extracellular region, extracellular matrix). The PANTHER Overrepresentation Test (FDR < 0.05) identified 10 significantly enriched BP terms (steroid metabolic process, protein targeting, protein transport, vesicle-mediated transport, intracellular protein transport, protein localization, transport, localization, single-multicellular organism process, multicellular organismal process), one MF term (SNAP receptor activity), and six CC terms (vesicle coat, SNARE complex, Golgi apparatus, cytoplasmic membrane-bounded vesicle, endoplasmic reticulum, cytoplasm).

All 236 annotated DEGs in the filtered list (log_2_ FC ≤ −1 or ≥ 1; adjusted *p*-value < 0.05) were submitted to the Reactome Pathways Analysis tool (Available online: https://reactome.org/PathwayBrowser/#TOOL=AT) together with their log_2_ FC values. Out of these, 132 were recognized by the Reactome database and considered for pathway analysis. All 23 pathways linked with the submitted genes with FDR < 0.05 are listed in Table 1. The table includes Reactome pathway identifiers, pathway name, the number of matched genes, plus the pathway expression representation, which was calculated from the average log_2_ FC of the matched genes. Overall, 17 pathways were upregulated, whereas another six were downregulated. The most highly upregulated pathway was ‘PERK regulates gene expression’ (R-HSA-381042) with an average log_2_ FC of 2.165, but the most downregulated pathway was ‘G0 and early G1′ (R-HSA-1538133) with an average log_2_ FC of −1.179. Table 2 lists pathways related to Wnt signalling, including their pathway’s identifiers, the number of matched genes, FDR values and pathway expression representation by an average of log_2_ FC.

#### 3.6.2. Control vs. TG_Monensin

**Figure 8:**
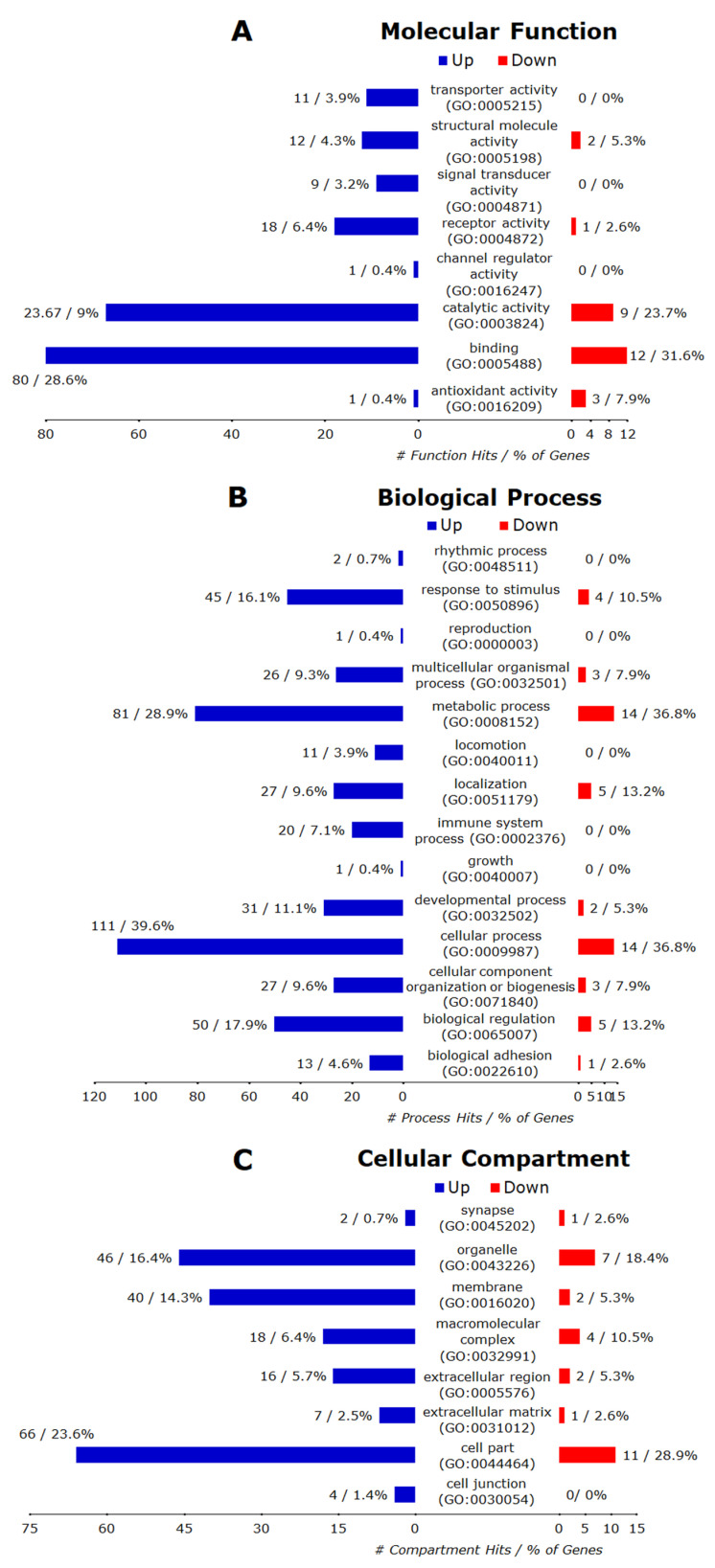
PANTHER GO-Slim gene ontology hits for significant DEGs due to *T. gondii* infection and treatment with monensin (TG_Monensin). (**A**) The down-regulated genes matched 3 MFs while the up-regulated genes matched seven MFs. The PANTHER Overrepresentation Test (FDR < 0.05) identified nine MFs down-regulated genes but none for up-regulated genes. (**B**) The down-regulated genes matched 10 BP terms while the up-regulated genes matched 11 terms. The PANTHER Overrepresentation Test (FDR < 0.05) identified 25 and nine overrepresented BPs for down- and up-regulated genes, respectively. (**C**) The down-regulated genes matched six CC terms while the up-regulated genes matched six CC terms. The PANTHER Overrepresentation Test (FDR < 0.05) identified 12 and nine overrepresented CCs for down-regulated and up-regulated genes, respectively.

Of 109 annotated downregulated genes, 101 were recognized by the PANTHER database and the hits for all GO annotations (BP, MF, CC) and their major categories were presented in Figure 8A–C, respectively. Overall, these downregulated genes matched 10 BP terms (biological adhesion, biological regulation, cellular component organization or biogenesis, cellular process, developmental process, localization, metabolic process, multicellular organismal process, reproduction, response to stimulus), three MF terms (binding, catalytic activity, structural molecule activity), and six CC terms (cell part, extracellular matrix, extracellular region, macromolecular complex, membrane, organelle). Further analysis with the PANTHER Overrepresentation Test (FDR < 0.05) revealed 25 significantly enriched BP terms (Table 3), nine significantly enriched MF terms (DNA binding, nucleic acid binding, DNA helicase activity, helicase activity, binding, single-stranded DNA binding, microtubule binding, microtubule motor activity, motor activity), and 12 significantly enriched CC terms (chromosome, nucleus, nuclear chromosome, organelle, protein-DNA complex, nucleoplasm, intracellular, cell part, microtubule, plasma membrane, membrane, cytoskeleton).

Of 201 annotated up-regulated genes, 173 were recognized by the PANTHER database and the hits for all GO annotations (BP, MF, CC) and their major categories are presented in Figure 8A–C, respectively. Overall, these up-regulated genes matched to 11 BP terms (biological regulation, cellular component organization or biogenesis, cellular process, developmental process, immune system process, localization, locomotion, metabolic process, multicellular organismal process, reproduction, response to stimulus), seven MF categories (antioxidant activity, binding, catalytic activity, receptor activity, signal transducer activity, structural molecule activity, transporter activity), and six CC terms (cell part, extracellular matrix, extracellular region, macromolecular complex, membrane, organelle). Further analysis with the PANTHER Overrepresentation Test (FDR < 0.05) found nine significantly enriched BPs (protein glycosylation, vesicle-mediated transport, protein transport, protein localization, intracellular protein transport, transport, localization, single-multicellular organism process, multicellular organismal process) and nine significantly enriched CC terms (vesicle coat, SNARE complex, Golgi apparatus, cytoplasmic membrane-bounded vesicle, endoplasmic reticulum, nuclear outer membrane-endoplasmic reticulum membrane network, cytoplasm, organelle, intracellular), but no significantly enriched MF terms.

All 310 DEGs with a symbol from the filtered list (log_2_ FC ≤ −1 or ≥ 1; adjusted *p*-value < 0.05) were submitted to the Reactome pathways analysis tool together with their log_2_ FC values. Out of these, only 180 were recognized by the Reactome database and considered for pathway analysis. Pathways linked with the submitted genes with FDR < 0.05 are listed in Table 4. Overall, the most up-regulated pathway was ‘PERK regulates gene expression’ (R-HSA-381042) with an average log_2_ FC of 2.065, whereas the most down-regulated pathway was ‘Resolution of D-loop structures through Holliday junction intermediates’ (R-HSA-5693568) and ‘Resolution of D-Loop structures’ (R-HSA-5693537), both with an average log_2_ FC of −1.239. Table 5 lists pathways related to Wnt signalling plus their pathway’s identifiers, the number of matched genes, FDR values, and pathway expression representation by an average of log_2_ FC.

#### 3.6.3. Control vs. TG_only

**Figure 9:**
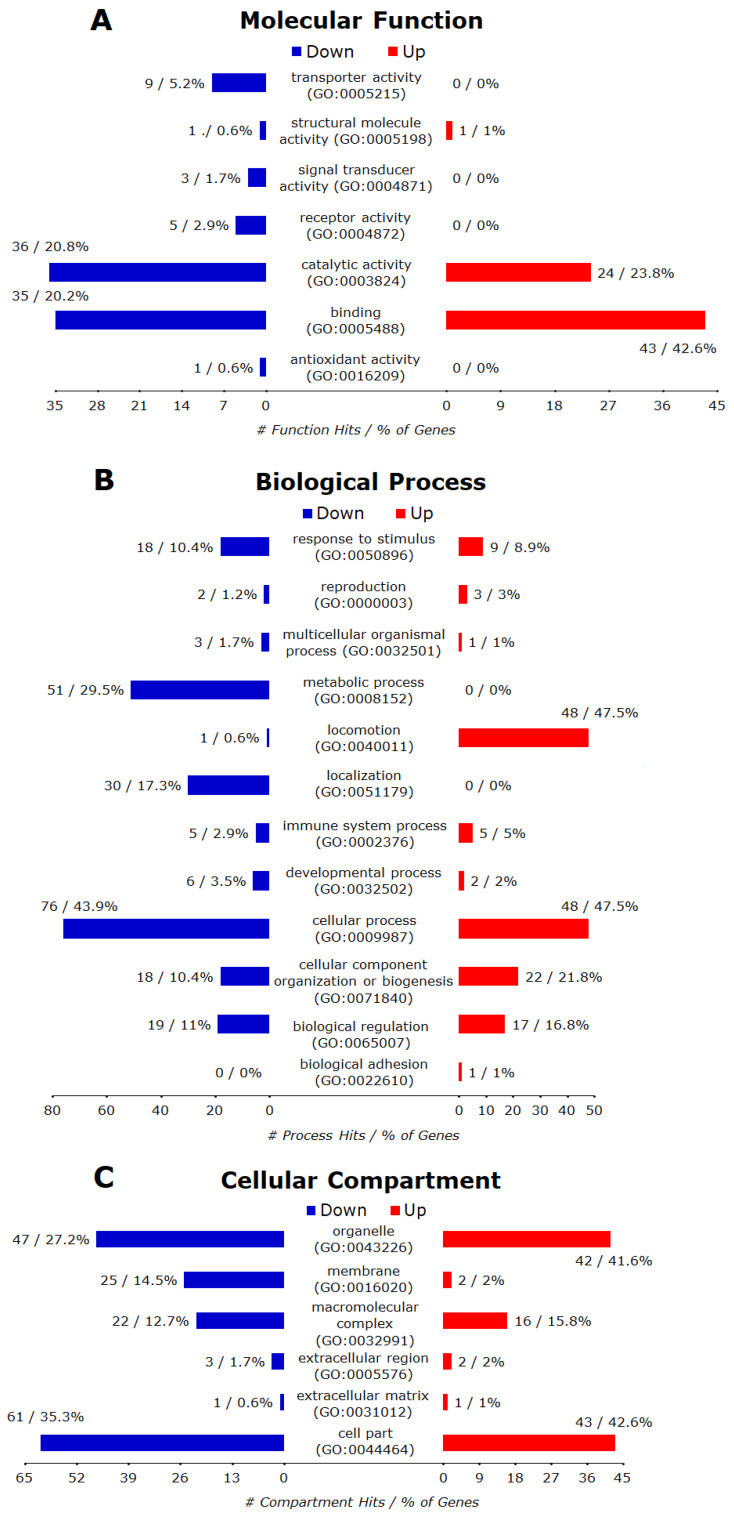
PANTHER GO-Slim gene ontology hits for significant DEGs due to *T. gondii* infection (TG_only). (**A**) The down-regulated genes matched five MF terms while the up-regulated genes matched eight MFs. No overrepresented MF was identified from the PANTHER Overrepresentation Test (FDR < 0.05) for both sets of genes. (**B**) The down-regulated genes matched nine PBs while the up-regulated genes matched 14 BPs. The PANTHER Overrepresentation Test (FDR < 0.05) identified two overrepresented BPs from up- but none for down-regulated genes. (**C**) The down-regulated genes matched seven CC terms while the up-regulated genes matched eight CC terms. No overrepresented CC was identified from the PANTHER Overrepresentation Test (FDR < 0.05) for both sets of genes.

Of 103 annotated down-regulated genes, only 38 were recognized by the PANTHER database, and the hits for all GO annotations (BP MF, CC) and their major categories were presented in Figure 9A–C. Overall, these upregulated genes matched to 9 BP terms (cellular process, metabolic process, localization, biological regulation, response to stimulus, cellular component organization or biogenesis, multicellular organismal process, developmental process, biological adhesion), five MF terms (binding, catalytic activity, transporter activity, signal transducer activity, receptor activity) and seven CC terms (cell part, organelle, macromolecular complex, membrane, extracellular region, synapse, extracellular matrix). Further analysis with the PANTHER Overrepresentation Test found no significantly enriched BP, MF or CC (FDR < 0.05).

Of 345 annotated up-regulated genes, only 280 were recognized by the PANTHER database and the hits for all GO annotations (BP MF, CC) and their major categories were presented in Figure 9A–C, respectively. Overall, these up-regulated genes matched to 14 BP terms (biological adhesion, biological regulation, cellular component organization or biogenesis, cellular process, developmental process, growth, immune system process, localization, locomotion, metabolic process, multicellular organismal process, reproduction, response to stimulus, rhythmic process), eight MF terms (antioxidant activity, binding, catalytic activity, channel regulator activity, receptor activity, signal transducer activity, structural molecule activity, transporter activity) and eight CC terms (cell junction, cell part, extracellular matrix, extracellular region, macromolecular complex, membrane, organelle, synapse). Further analysis with the PANTHER Overrepresentation Test found BP to be significantly enriched, but there were no significantly enriched MF or CC (FDR < 0.05).

All 448 DEGs with a symbol from the filtered list (log_2_ FC ≤ −1 or ≥ 1; adjusted *p* value < 0.05) were submitted to Reactome’s ‘Pathways Analysis’ tool together with their log_2_ FC values. Out of these, only 232 were recognized by the Reactome database and considered for pathway analysis. Pathways linked to the submitted genes with FDR < 0.05 are listed in Table 6. Surprisingly, even though there were more genes submitted compared to Monensin_only, only four pathways passed the corrected overrepresentation probability test (FDR < 0.05). All four pathways were upregulated, with the most highly upregulated pathway being ‘Interferon alpha/beta signalling’ (R-HSA-909733) with an average log_2_ FC of 1.57. Table 7 lists pathways related to the Wnt signalling plus their pathway’s identifiers, the number of matched genes, FDR values, and pathway expression representation by an average of log_2_ FC.

## 4. Discussion

In this study, we utilized microarray analysis to evaluate the transcriptome profile of *T. gondii*-infected hBMECs treated with the Wnt inhibitor monensin. First, we investigated the effect of Wnt inhibition by monensin on the in vitro growth of *T. gondii* inside hBMECs and microglia cells. *T. gondii* growth inhibition was evidenced by the significant reduction in the number of tachyzoites (Figure 1) and the relative similarity in cell density levels (Figure 2) between infected and non-infected cultures. Our data showed that parasite inhibition increased as the concentration of monensin increased (i.e. dose-dependent) and that monensin at 0.1 µM concentration significantly decreased the growth of *T. gondii* tachyzoites. Consistent with this, it has been reported that monensin reduced *T. gondii* growth in human foreskin fibroblasts [26,27]. Our result also fits within the effective range of monensin concentrations (0.01 and 0.1 µM) reported against human cytomegalovirus (HCMV) [14]. Interestingly, monensin was more effective in inhibiting *T. gondii* growth in hBMECs compared to microglia cells. Moreover, in the presence of monensin treatment, infected and noninfected hBMECs exhibited significant reduction in their growth rates. However, the differential variation in the magnitude of cell growth reduction between the two cell lines suggests that monensin had more cytotoxic effect on hBMECs compared to microglia cells. These findings were the basis for choosing hBMECs to determine the effects of monensin on host gene transcription in the context of *T. gondii* infection.

Next, we determined the effect of *T. gondii* with or without monensin treatment on the integrity of cerebrovascular endothelial cell monolayer. Our findings showed that *T. gondii* infection can compromise the integrity of BBB endothelial cells compared to uninfected cells (*p* < 0.05; Figure 3). Consistent with our finding, *T. gondii* was reported to compromise the cell integrity to facilitate a paracellular migration route across the BBB [28]. Cellular barrier function is mediated through intercellular tight junction (TJ) proteins, which can be dysregulated by *T. gondii* infection to increase barrier permeability and parasite crossing [29]. Alteration in the TJ function may occur either through a direct effect of *T. gondii* infection on the host cell cytoskeleton [30] or via the indirect action of *T. gondii*-induced proinflammatory cytokines, which can promote the opening of TJ that would in turn allow leukocytes to migrate across the endothelial barrier into cerebral tissue [31,32]. *T. gondii*-infected and treated hBMECs seemed to maintain their resistance, presumably due to the inhibitory effect of monensin on *T. gondii* growth, thereby protecting cells from cytoskeletal rearrangement associated with parasite invasion and growth, which compromises TJ and BBB integrity. Given the ability of this parasite to invade and use leukocytes as a Trojan-horse to migrate across the BBB [33], further studies should investigate how monensin may influence the effect of *T. gondii* on leukocytes’ ability to cross the BBB. If monensin is effective in inhibiting *T. gondii* growth inside leukocytes, then this may thwart the parasite’s ability to disseminate to the CNS tissue.

The exact mechanism of anti-*T. gondii* activity of monensin is unknown, however this compound seems to exert its action by directly interfering with one or more functions in the parasite. For example, osmotic swelling of the parasite [34], presumably due to its inability to regulate ionic homeostasis as a result of Na^+^ influx [35], interference with vesicular trafficking [36], altering the parasite’s cell cycle [26], or inducing nutrient stress and TgMSH-1-mediated autophagy [27], have all been suggested to contribute to *T. gondii* death. Interestingly, pre-treatment of hBMECs with monensin inhibited the susceptibility of hBMEC to gram-negative bacteria *Citrobacter freundii* [37] and *Brucella abortus* [38] invasion or survival in a dose-dependent manner. Monensin exhibits potent anticancer activity on several types of cancer, by inhibiting cell proliferation, cell cycle progression, and cell migration and by inducing apoptosis [39]. Monensin may also accomplish its anticancer effect by targeting multiple signaling pathways, particularly the epidermal growth factor receptor (EGFR) and Wnt signaling pathways [39,40]. Given these pervasive effects of monensin, it is possible that monensin exerts its anti-*T. gondii* effect, by altering the host response to infection in addition to its direct antiparasitic effect.

Therefore, we investigated whether the secondary effects of monensin on the host cells may contribute to parasite clearance. We performed microarray analysis to test the hypothesis that treatment with 0.1 µM monensin modulates signalling pathways, such as the Wnt pathway, which in turn inhibits *T. gondii* infection and enhances hBMECs survival. The microarray quality control checks indicated that the datasets had acceptable quality. Variations within samples were minimal (Figure 4) and variations between samples were made comparable by RMA normalization (Figure S1). Our analysis revealed 480 DEGs in hBMECs treated with monensin compared to control untreated hBMECs. Since monensin induced significant changes in the gene transcription of treated cells, we wanted to investigate whether monensin also can cause the same changes in the presence of *T. gondii* infection. Interestingly, 424 DEGs were detected in *T. gondii*-infected hBMECs treated with monensin compared to control hBMECs. When we compared DEGs in monensin-treated uninfected cells to DEGs in monensin-treated *T. gondii*-infected cells, different levels of expression of specific genes were detected between the two groups, showing the differential response of treated cells in the presence or absence of infection. This finding is anticipated because a subset of these DEGs can be expected to be mechanistically involved in the infection process and thus their expression is unlikely to change in response to treatment per se. Interestingly, the TG_only group expression profile was distinct from the expression profiles of the other three groups (TG_Monensin group, Monensin_only group and Control_only group) as shown in the dendrogram (Figure 4A). This clustering is supported by the detection of 902 DEGs in hBMECs infected by *T. gondii* without monensin treatment (TG_only group), compared to 424 DEGs detected in infected and treated cells (TG_Monensin group). Such a significantly large number of DEGs in the TG_only group indicates that monensin must have attenuated the adverse impact of *T. gondii* infection on the treated cells, probably by interfering with signalling pathways essential for the parasite growth or via direct inhibition of the parasite proliferation, providing further support to the antiparasitic effect of monensin observed in Figure 1 and Figure 2.

PANTHER GO enrichment analysis, used to functionally annotate all DEGs in each group, revealed similar terms in the three GO categories between the examined groups. Reactome pathway enrichment analysis of the DEGs of hBMECs treated with monensin revealed that monensin treatment downregulated pathways related to steroids, cholesterol and protein biosynthesis and their transport between endoplasmic reticulum (ER) and Golgi apparatus. Transcriptional analysis of *T. gondii*-infected hBMECs treated with monensin revealed a similar transcriptional pattern to the Monensin_only group. These results suggest that parasite inhibition may have been achieved through dysregulation of the biosynthesis of key nutrients in the host cells and/or inhibition of nutrient transport between cell organelles, limiting their transport into parasitophorous vacuole. This finding is consistent with a previous transcriptomic study of monensin-treated porcine kidney (PK)-15 cells, which revealed an overrepresentation of the downregulated genes involved in the biosynthesis of spliceosome, ribosome, and protein processing in ER, suggesting that monensin, via down-regulation of protein biosynthesis, can limit the parasite growth and proliferation [41].

Our data also showed that monensin administration modulated pathways related to DNA replication in the nucleus and cell cycle. In our study, we have identified 39 significantly upregulated pathways related to DNA replication, cell cycle and cell division in the TG_Monensin group compared to only three significantly upregulated pathways from the Monensin_only transcriptome data. The significantly increased number of these upregulated pathways in the TG_Monensin group is probably attributed to the dual effects of monensin and parasite infection on the host cell transcriptome. The effects of monensin on DNA synthesis, and repair of the parasite and the host cells, can be also inferred from the downregulated pathways identified in both the Monensin_only and the TG_Monensin groups. Some of the downregulated pathways were related to DNA repair mechanisms, such as resolution of D-loop structures through Holliday junction intermediates (R-HSA-5693568), resolution of D-loop structures (R-HSA-5693537), resolution of D-loop structures through synthesis-dependent strand annealing (R-HSA-5693554), and unwinding of DNA (R-HSA-176974). In addition, key DNA repair mechanisms related to molecular functions, such as DNA helicase activity (GO:0003678), helicase activity (GO:0004386), kinase regulator activity (GO:0019207), nucleic acid binding (GO:0003676) and binding (GO:0005488), were also identified as overrepresented (PANTHER Overrepresentation Test) from downregulated genes of the Monensin_only group.

Interestingly, both the Monensin_only and the TG_Monensin groups downregulated cell cycle-related pathways (e.g. cell cycle (R-HSA-1640170); G0 and early G1 (R-HSA-1538133) and transcription of E2F targets under negative control by p107/RBL1 and p130/RBL2 in complex with HDAC1 [R-HSA-1362300]) and upregulated PERK regulates gene expression (R-HSA-381042), which leads to cell cycle arrest in host cells. Monensin has been shown to arrest the parasite cell cycle process [26]. Thus, we can infer that monensin can inhibit both parasite and host cell cycle processes that ultimately lead to growth inhibition of *T. gondii*. This finding is consistent with the upregulation of the DNA damage-inducible transcript 3 (*DDIT3*), a key gene in the upregulated pathway, which encodes a stress-inducible transcription factor that induces cell cycle arrest and apoptosis in mammalian cells. Our finding is consistent with a previous study that showed that monensin induced *DDIT3*, a key inhibitor of the Wnt signalling pathway [42], in prostate cancer cells [43]. The key genes identified in those downregulated pathways are *CCNA2*, that controls both the G1/S and the G2/M transition phases of the cell cycle, and *MYBL2*, that functions as a physiological regulator of cell cycle progression. While there are no published data that show the inhibitory effect of monensin on *CCNA2*, there are reports on monensin as a potent inhibitor of the *MYB* gene family that includes *MYBL1* and *MYBL2* [44,45,46].

The link between *T. gondii* and the Wnt signalling pathway has been already established. In one study, the Wnt pathway was found to be upregulated during *T. gondii* infection in brain cells [47]. In another study, the virulence factor ROP18 of *T. gondii* was found to inhibit the Wnt signalling pathway in neural stem cells [48]. In the present study, Wnt signalling and Wnt-related pathways were not significantly affected by treatment with 0.1 µM monensin, rejecting the hypothesis that inhibition of *T. gondii* growth is largely mediated by the aberrant Wnt signalling in the host cells due to monensin treatment. All Wnt-related genes, such as Frizzled receptors, beta-catenin and protein kinase C (PKC) had modest expression after treatment. The same also applies to the transcriptome of TG_only and TG_Monensin groups. This finding suggests that monensin treatment did not affect the Wnt signalling pathway in hBMECs and that inhibition of *T. gondii* infection in hBMECs following monensin treatment was not achieved through direct blocking of the Wnt signalling pathway. Our study did however identify changes in the expression of genes related to other signal pathways involved in cell cycle control, apoptosis, and DNA synthesis and repair. These data further support the pleiotropic effects of monensin on multiple biological processes.

We were also interested in comparing host gene expression in uninfected to *T. gondii*-infected hBMECs in the absence of monensin treatment to better understand the types of host responses induced by infection. The transcriptome analysis of hBMECs infected with *T. gondii* for 24 h (TG_only) revealed significantly increased interferon-alpha/beta and gamma signalling-related pathways. This finding is consistent with previous transcriptome studies in murine macrophages [49] and human fibroblast cells [50], reaffirming the deregulation of the immune response following *T. gondii* infection of hBMECs. The increased expression of the *MT1M* gene was also reported previously [51], where *MT1M* upregulation has been suggested to contribute to the cellular homeostasis of transition metals, particularly zinc, during *T. gondii* infection. The down-regulation of microRNA 29a has also been reported in a mouse brain infected by the cystogenic strain (type II) [52], which is less virulent than the RH (type I) strain used in our study, suggesting that inhibition of microRNA 29a is common in *T. gondii* infection, independent of the parasite virulence.

## 5. Conclusions

We showed that the ionophore monensin, an inhibitor of the Wnt pathway, had a significant anti-*T. gondii* activity against the tachyzoite stage of *T. gondii* grown in hBMECs and microglia in vitro at 0.1 µM concentration. We also showed an association between *T. gondii* infection and compromisation of the hBMEC integrity, which was restored by treatment with 0.1 µM monensin. Transcriptome analysis provided evidence that treatment of hBMECs with monensin is accompanied by reduced expression of genes in pathways involved in steroids, cholesterol and protein biosynthesis and their transport between ER and Golgi apparatus. These data indicate that inhibition of *T. gondii* infection in hBMECs was not directly achieved through blocking Wnt signalling and that anti-*T. gondii* activity of monensin appears to be mediated independently of the Wnt pathway. Rather, our findings support the notion that parasite inhibition was in part due to inhibition of transport between organelles within cells and limiting nutrient transport into parasitophorous vacuole. In addition, monensin seems to inhibit the parasite growth through interference with cell cycle and DNA synthesis and repair mechanisms. More studies to elucidate how host-targeted effects of monensin on hBMECs inhibits *T. gondii* infection are warranted. Future work should also include experiments on the protein level to assess whether differential gene regulation is paralleled by similar proteomic changes.

## Figures and Tables

**Table 1:** All 23 Reactome pathways affected by 24 h of 0.1 μM monensin treatment (Monensin_only) with FDR < 0.05.

Pathway ID	Pathway Name	Genes Found/Total	Average Log_2_ FC
R-HSA-381119	Unfolded protein response (UPR)	31/160	1.440
R-HSA-381070	IRE1alpha activates chaperones	24/110	1.273
R-HSA-381038	XBP1(S) activates chaperone genes	21/106	1.293
R-HSA-1655829	Regulation of cholesterol biosynthesis by SREBP (SREBF)	19/86	1.279
R-HSA-2426168	Activation of gene expression by SREBF (SREBP)	16/70	1.295
R-HSA-199977	ER to Golgi anterograde transport	17/163	1.318
R-HSA-8957322	Metabolism of steroids	24/321	1.302
R-HSA-446203	Asparagine N-linked glycosylation	26/420	1.301
R-HSA-381183	ATF6 (ATF6-alpha) activates chaperone genes	6/15	1.766
R-HSA-381033	ATF6 (ATF6-alpha) activates chaperones	6/17	1.766
R-HSA-6807878	COPI-mediated anterograde transport	12/106	1.316
R-HSA-948021	Transport to the Golgi and subsequent modification	17/218	1.318
R-HSA-191273	Cholesterol biosynthesis	10/72	1.373
R-HSA-392499	Metabolism of proteins	75/2432	0.969
R-HSA-204005	COPII-mediated vesicle transport	9/76	1.381
R-HSA-6811442	Intra-Golgi and retrograde Golgi-to-ER traffic	14/217	1.127
R-HSA-176974	Unwinding of DNA	4/12	−1.121
R-HSA-381042	PERK regulates gene expression	6/38	2.165
R-HSA-1362300	Transcription of E2F targets under negative control by p107 (RBL1) and p130 (RBL2) in complex with HDAC1	4/20	−1.096
R-HSA-1538133	G0 and early G1	5/38	−1.179
R-HSA-1640170	Cell cycle	25/681	−0.763
R-HSA-6811438	Intra-Golgi traffic	5/48	1.558
R-HSA-5693554	Resolution of D-loop structures through synthesis-dependent strand annealing (SDSA)	4/30	−1.129

Note: Greyed rows indicate down-regulated pathways.

**Table 2:** Differentially expressed genes related to Wnt signaling (Monensin_only).

Pathway ID	Pathway Name	Entities Found/Total	Average Log_2_ FC	FDR Values
R-HSA-201681	TCF-dependent signalling in response to Wnt	2/216	1.232	0.918
*Matched Genes*	HIST1H2AJ; HIST1H3F			
R-HSA-195721	Signalling by Wnt	2/330	1.232	0.987
*Matched Genes*	HIST1H2AJ; HIST1H3F			

**Table 3:** All 25 significantly enriched biological processes from 101 annotated upregulated genes, sorted with the most significant at the top (TG_only).

PANTHER GO-Slim Biological Process (PO)	No. of Matched Genes	FDR
DNA metabolic process (GO:0006259)	23	5.44 × 10^−16^
Nucleobase-containing compound metabolic process (GO:0006139)	38	1.04 × 10^−7^
Cell cycle (GO:0007049)	19	1.22 × 10^−7^
Chromosome segregation (GO:0007059)	9	1.51 × 10^−7^
Mitosis (GO:0007067)	11	1.1 × 10^−6^
DNA repair (GO:0006281)	9	6.88 × 10^−6^
Organelle organisation (GO:0006996)	21	1.04 × 10^−5^
Regulation of cell cycle (GO:0051726)	9	1.23 × 10^−5^
DNA replication (GO:0006260)	8	3.44 × 10^−5^
Nitrogen compound metabolic process (GO:0006807)	30	4.27 × 10^−5^
Chromatin organisation (GO:0006325)	9	1.87 × 10^−4^
Metabolic process (GO:0008152)	48	6.35 × 10^−4^
Primary metabolic process (GO:0044238)	41	1.18 × 10^−3^
DNA recombination (GO:0006310)	4	2.59 × 10^−3^
Cellular component organisation (GO:0016043)	22	3.08 × 10^−3^
Cell communication (GO:0007154)	2	3.69 × 10^−3^
Cellular component organisation or biogenesis (GO:0071840)	22	5.55 × 10^−3^
Regulation of gene expression, epigenetic (GO:0040029)	4	6.23 × 10^−3^
Chromatin assembly (GO:0031497)	3	0.0146
Signal transduction (GO:0007165)	2	0.0147
Meiosis (GO:0007126)	3	0.0433
Multicellular organismal process (GO:0032501)	1	0.0459
Single-multicellular organism process (GO:0044707)	1	0.0469
Cell surface receptor signalling pathway (GO:0007166)	0	0.0485
Cell proliferation (GO:0008283)	3	0.0487

**Table 4:** The top 30 out of 55 Reactome pathways affected by *T. gondii* infection and treatment with 0.1 μM monensin (TG_Monensin). Among the 310 genes submitted, only 180 were recognized by the Reactome database and considered for analysis.

Pathway ID	Pathway Name	No. of Matched Genes	Average Log_2_ FC
R-HSA-381038	XBP1(S) activates chaperone genes	27/106	1.399
R-HSA-381070	IRE1alpha activates chaperones	30/110	1.373
R-HSA-381119	Unfolded protein response (UPR)	38/160	1.469
R-HSA-69278	Cell cycle, mitotic	43/569	−1.000
R-HSA-1640170	Cell cycle	47/681	−1.017
R-HSA-1538133	G0 and early G1	10/38	−1.091
R-HSA-446203	Asparagine N-linked glycosylation	31/420	1.307
R-HSA-199977	ER to Golgi anterograde transport	18/163	1.343
R-HSA-453279	Mitotic G1-G1/S phases	17/173	−1.091
R-HSA-69620	Cell cycle Checkpoints	22/279	−1.017
R-HSA-381183	ATF6 (ATF6-alpha) activates chaperone genes	6/15	1.612
R-HSA-6811442	Intra-Golgi and retrograde Golgi-to-ER traffic	19/217	0.809
R-HSA-6807878	COPI-mediated anterograde transport	13/106	1.362
R-HSA-381033	ATF6 (ATF6-alpha) activates chaperones	6/17	1.612
R-HSA-392499	Metabolism of proteins	92/2432	0.796
R-HSA-948021	Transport to the Golgi and subsequent modification	18/218	1.343
R-HSA-1362300	Transcription of E2F targets under negative control by p107 (RBL1) and p130 (RBL2) in complex with HDAC1	6/20	−1.054
R-HSA-6811434	COPI-dependent Golgi-to-ER retrograde traffic	12/106	0.447
R-HSA-141424	Amplification of signal from the kinetochores	11/94	−0.858
R-HSA-141444	Amplification of signal from unattached kinetochores via a MAD2 inhibitory signal	11/94	−0.858
R-HSA-69618	Mitotic spindle checkpoint	11/110	−0.858
R-HSA-68886	M Phase	23/389	−0.912
R-HSA-2500257	Resolution of sister chromatid cohesion	12/133	−0.872
R-HSA-5693568	Resolution of D-loop structures through Holliday junction intermediates	6/36	−1.239
R-HSA-8856688	Golgi-to-ER retrograde transport	12/147	0.447
R-HSA-5693537	Resolution of D-Loop structures	6/37	−1.239
R-HSA-381042	PERK regulates gene expression	6/38	2.065
R-HSA-69206	G1/S Transition	12/150	−1.103
R-HSA-1362277	Transcription of E2F targets under negative control by DREAM complex	5/25	−1.070
R-HSA-774815	Nucleosome assembly	7/54	−1.108

**Table 5:** Expressed genes related to Wnt signaling (TG_Monensin) according to the Reactome classification.

Pathway ID	Pathway Name	Genes Found/Total	Average Log_2_ FC	FDR Values
R-HSA-195721	Signalling by Wnt	5/330	−1.207	0.911
*Matched Genes*	HIST1H2BM; HIST1H2AJ; HIST1H3F; HIST1H3G; HIST1H2BE; HIST1H3B; HIST1H2AB			
R-HSA-201681	TCF-dependent signalling in response to Wnt	5/216	−1.207	0.617
*Matched Genes*	HIST1H2BM; HIST1H2AJ; HIST1H3F; HIST1H3G; HIST1H2BE; HIST1H3B; HIST1H2AB			

Note: HIST1H2BM/HIST1H2BE and HIST1H3B/HIST1H3F are duplicated genes.

**Table 6:** Reactome pathways affected by *T. gondii* infection (TG_only) with FDR < 0.05.

Pathway ID	Pathway Name	Genes Found/Total	Average Log_2_ FC
R-HSA-909733	Interferon alpha/beta signalling	38/184	1.611
R-HSA-913531	Interferon Signalling	50/388	1.532
R-HSA-1280215	Cytokine Signalling in the Immune system	72/1051	1.535
R-HSA-877300	Interferon gamma signalling	23/250	1.359

**Table 7:** Differentially expressed genes involved in Wnt signaling (TG_only) according to the Reactome classification.

Pathway ID	Pathway Name	Genes Found/Total	Average Log_2_ FC	FDR Values
R-HSA-195721	Signalling by Wnt	7/330	1.303	0.937
*Matched Genes*	HIST1H4B; FZD7; HIST1H2BG; HIST2H3D; PLCB1; GNG11; HIST1H2AB			
R-HSA-201681	TCF-dependent signalling in response to Wnt	4/216	1.279	0.939
*Matched Genes*	HIST1H4B; HIST1H2BG; HIST2H3D; HIST1H2AB			
R-HSA-3858494	Beta-catenin independent Wnt signalling	3/165	1.335	0.923
*Matched Genes*	FZD7; PLCB1; GNG11			

## Supplementary Materials

The following are available online at https://www.mdpi.com/2076-2607/8/6/842/s1, Figure S1. Raw and normalized expression measurements of the microarray data. Box and whiskers plots showing expression values of all samples pre- (A) and post-normalization (B). The plots consist of boxes with a central horizontal line that represents the median of the data and two tails (dotted vertical lines) that represent the upper and lower quartile. The variable lengths of the solid vertical lines show the heterogeneity in the distribution of the microarray files before implementing normalization. The samples were normalized using the widely used robust multiarray average (RMA) method, which considers background correction and variation between chipsets [21,22]. Normalization improved the overall distribution between samples and rendered the data more balanced for the accurate sample to sample comparison ahead of the analysis, Table S1. List of 19 DEGs induced in three groups (Monensin_only, TG_Monensin, and TG_only) compared to the control group, Table S2. The top 30 most significant DEGs detected in hBMECs after 24 h of 0.1 µM monensin treatment (Monensin_only), Table S3. The top 30 upregulated genes detected in hBMECs treated with 0.1 μM monensin (Monensin_only) sorted in a descending order, with the highest log_2_ FC at the top, Table S4. The top 30 downregulated genes detected in hBMECs treated with 0.1 μM monensin (Monensin_only) sorted with the lowest log_2_ FC at the top, Table S5. The top 30 most significant DEGs detected in hBMECs infected with *T. gondii* and treated with 0.1 μM monensin (TG_Monensin), Table S6. The top 30 upregulated genes detected in 0.1 μM monensin treated and *T. gondii*-infected hBMECs (TG_Monensin) sorted with the highest log_2_ FC values at the top, Table S7. The top 30 downregulated genes detected in 0.1 μM monensin treated and *T. gondii*-infected hBMECs (TG_Monensin) sorted with the lowest log_2_ FC values at the top, Table S8. The top 30 most significant DEGs induced in hBMECs 24 h after *T. gondii* infection (TG_only), Table S9. The top 30 upregulated genes detected in *T. gondii*-infected hBMECs (TG_only) sorted with the highest log_2_ FC at the top, Table S10. The top 30 downregulated genes detected in *T. gondii*-infected hBMECs (TG_only) sorted with the lowest log_2_ FC at the top.
