# Supplementary material for: Transcriptome Profiling of Toxoplasma gondii-Infected Human Cerebromicrovascular Endothelial Cell Response to Treatment with Monensin
Source: Microorganisms. 2020 Jun 4;8(6):842. doi: 10.3390/microorganisms8060842 (PMC7356316; doi:10.3390/microorganisms8060842)
Supplement: Supplementary file 1 [file microorganisms-08-00842-s001.pdf]

Supplementary files

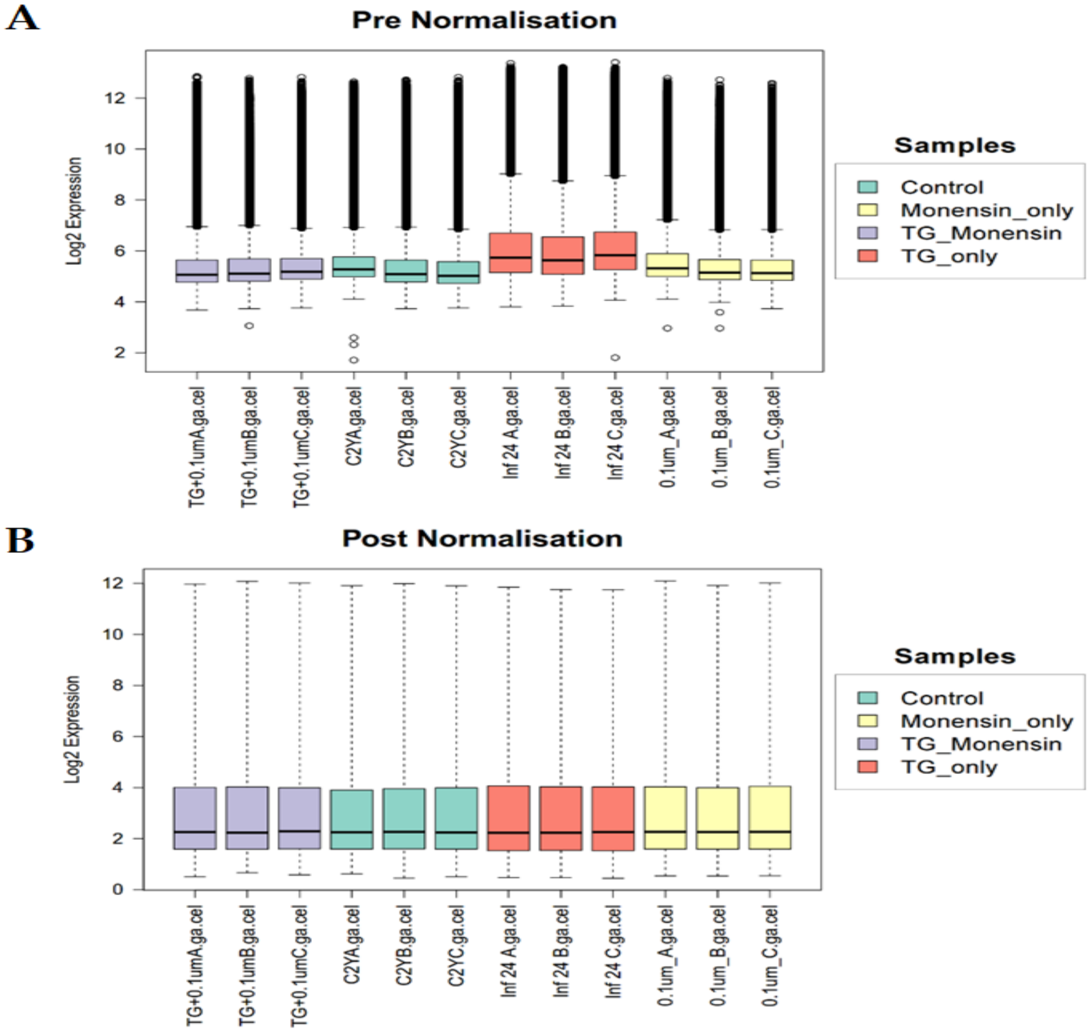

Figure S1. Raw and normalized expression measurements of the microarray data. Box and whiskers plots showing expression values of all samples pre- (**A**) and post-normalization (**B**). The plots consist of boxes with a central horizontal line that represents the median of the data and two tails (dotted vertical lines) that represent the upper and lower quartile. The variable lengths of the solid vertical lines show the heterogeneity in the distribution of the microarray files before implementing normalization. The samples were normalized using the widely used robust multiarray average (RMA) method, which considers background correction and variation between chipsets [21, 22]. Normalization improved the overall distribution between samples and rendered the data more balanced for the accurate sample to sample comparison ahead of the analysis.

Table S1. List of 19 DEGs induced in three groups (Monensin_only, TG_Monensin, and TG_only) compared to the control group.

Gene Cluster ID	Gene Symbol	Gene Description	Log$_2$ FC		
			Monensin_only	TG_Monensin	TG_only
16995890	HMGCS1	3-hydroxy-3-methylglutaryl-CoA synthase 1	-1.692	-1.271	1.393
17119762	NA	NA	2.288	1.028	2.335
17120004	NA	NA	-1.742	-1.149	1.048
16982145	NA	NA	-1.470	-1.060	1.484
17058000	NA	NA	-1.352	-1.111	1.067
17100677	NA	NA	2.311	1.061	1.897
16736638	E2F8	E2F transcription factor 8	1.399	1.428	1.228
16653585	NA	NA	-1.975	-1.254	-1.705
17100655	NA	NA	2.780	1.021	1.343
16655751	NA	NA	-1.464	-1.258	-1.165
17117424	NA	NA	-1.198	-1.276	1.245
16719973	NA	NA	-1.158	-1.035	-1.155
17126272	NA	NA	2.288	1.028	2.335
16690511	TAF13	TATA-box binding protein associated factor 13	-2.310	-2.186	-1.161
17072622	LOC101927657	Uncharacterized LOC101927657	1.078	1.371	1.406
16864907	ERVV-2	Endogenous retrovirus group V member 2	-2.030	-1.026	-1.007
16911236	NA	NA	1.267	1.522	1.207
16677898	LOC100287497	Septin 7 pseudogene	-1.467	-1.334	1.672
17100685	NA	NA	3.600	1.743	1.222

Table S2. The top 30 most significant DEGs detected in hBMECs after 24 hours of 0.1 μM monensin treatment (Monensin_only).

Gene Cluster ID	Gene Symbol	Gene Description	Log_2 FC
**17100663**	NA	NA*	-2.527
**17100655**	NA	NA	-2.780
**17100695**	NA	NA	-2.104
**16766578**	DDIT3	DNA damage inducible transcript 3	2.984
**17100675**	NA	NA	-2.761
**16819325**	HERPUD1	Homocysteine inducible ER protein with ubiquitin-like domain 1	2.052
**17100685**	NA	NA	-3.600
**17126276**	NA	NA	-1.988
**17126264**	NA	NA	-2.090
**16835125**	GOSR2	Golgi SNAP receptor complex member 2	1.927
**16979256**	SEC24D	SEC24 homolog D, COPII coat complex component	1.273
**17100693**	NA	NA	-1.753
**16679936**	NA	NA	-1.675
**17066561**	POLR3D	RNA polymerase III subunit D	1.713
**17100679**	NA	NA	-2.751
**16941167**	MANF	Mesencephalic astrocyte derived neurotrophic factor	1.944
**16949292**	DNAJB11	DnaJ heat shock protein family (Hsp40) member B11	1.441
**17014309**	ACAT2	Acetyl-CoA acetyltransferase 2	1.241
**16975954**	SCFD2	Sec1 family domain containing 2	1.811
**16725041**	FAM111B	Family with sequence similarity 111 member B	-2.393
**16690511**	TAF13	TATA-box binding protein associated factor 13	2.310
**16912975**	ACSS2	Acyl-CoA synthetase short-chain family member 2	1.312
**17100691**	NA	NA	-1.628
**16957771**	TMEM39A	Transmembrane protein 39A	1.350
**16820076**	TMEM208	Transmembrane protein 208	1.331
**16995890**	HMGCS1	3-hydroxy-3-methylglutaryl-CoA synthase 1	1.692
**17012079**	NUS1	NUS1 dehydrodolichyl diphosphate synthase subunit	1.207
**16917054**	LRRN4	Leucine rich repeat neuronal 4	-1.453
**17090161**	GPR107	G protein-coupled receptor 107	1.461
**16983742**	NA	NA	-2.235

* Twelve DEGs are indicated as 'NA' (not available) as they do not have annotated gene symbols because they are either unpublished or uncharacterized non-coding RNA genes.

Table S3. The top 30 upregulated genes detected in hBMECs treated with 0.1 μM monensin (Monensin_only) sorted in a descending order with the highest $\log_2$ FC at the top.

Gene Cluster ID	Gene Symbol	Gene Description	$\log_2$ FC
16766578	DDIT3	DNA damage inducible transcript 3	2.984
16690511	TAF13	TATA-box binding protein associated factor 13	2.310
16819325	HERPUD1	Homocysteine inducible ER protein with ubiquitin-like domain 1	2.052
16864907	ERVV-2	Endogenous retrovirus group V member 2, envelope	2.030
16774188	COG6	Component of oligomeric Golgi complex 6	1.982
16898890	MCEE	Methylmalonyl-CoA epimerase	1.954
16800301	PDIA3	Protein disulfide isomerase family A member 3	1.950
16941167	MANF	Mesencephalic astrocyte derived neurotrophic factor	1.944
16835125	GOSR2	Golgi SNAP receptor complex member 2	1.927
16868827	YIPF2	Yip1 domain family member 2	1.876
16673104	HSD17B7	Hydroxysteroid 17-beta dehydrogenase 7	1.865
16659133	NPPA-AS1	NPPA antisense RNA 1	1.855
16975954	SCFD2	Sec1 family domain containing 2	1.811
17103946	GPR173	G protein-coupled receptor 173	1.754
17066561	POLR3D	RNA polymerase III subunit D	1.713
16995890	HMGCS1	3-hydroxy-3-methylglutaryl-CoA synthase 1	1.692
16900053	EIF2AK3	Eukaryotic translation initiation factor 2 alpha kinase 3	1.689
17000518	HSPA9	Heat shock protein family A (Hsp70) member 9	1.649
16704055	HSD17B7P2	Hydroxysteroid 17-beta dehydrogenase 7 pseudogene 2	1.600
16772733	GOLGA3	Golgin A3	1.595
16700872	LYST	Lysosomal trafficking regulator	1.574
16927633	SDF2L1	Stromal cell-derived factor 2 like 1	1.570
16950686	SEC13	SEC13 homolog, nuclear pore and COPII coat complex component	1.565
16708161	ENTPD7	Ectonucleoside triphosphate diphosphohydrolase 7	1.556
16659102	CLCN6	Chloride voltage-gated channel 6	1.545
16941052	CYB561D2	Cytochrome b561 family member D2	1.532
16848079	WIPI1	WD repeat domain, phosphoinositide interacting 1	1.527
16829139	MVD	Mevalonate diphosphate decarboxylase	1.520
16842576	IFT20	Intraflagellar transport 20	1.494
16774551	COG3	Component of oligomeric Golgi complex 3	1.471

Table S4. The top 30 downregulated genes detected in hBMECs treated with 0.1 μM monensin (Monensin_only) sorted with the lowest $\log_2$ FC at the top.

Gene Cluster ID	Gene Symbol	Gene Description	$\mathbf{Log_2}$ FC
16798216	SNORD116-24	Small nucleolar RNA, C/D box 116-24	-2.543
16725041	FAM111B	Family with sequence similarity 111 member B	-2.393
17093722	RMRP	RNA component of mitochondrial RNA processing endoribonuclease	-1.716
17122086	LINC01224	Long intergenic non-protein coding RNA 1224	-1.555
17079293	CCNE2	Cyclin E2	-1.511
16714747	RTKN2	Rhotekin 2	-1.511
17016499	HIST1H1B	Histone cluster 1 H1 family member b	-1.511
16986913	VCAN	versican	-1.504
17080595	DSCC1	DNA replication and sister chromatid cohesion 1	-1.488
16917054	LRRN4	Leucine rich repeat neuronal 4	-1.453
16671514	MIR4258	MicroRNA 4258	-1.418
16732891	NRGN	Neurogranin	-1.413
17121990	ZNF730	Zinc finger protein 730	-1.399
16736638	E2F8	E2F transcription factor 8	-1.399
17004903	EDN1	Endothelin 1	-1.361
17020019	MCM3	Minichromosome maintenance complex component 3	-1.323
16919769	NCOA5	Nuclear receptor coactivator 5	-1.321
17016490	HIST1H2AJ	Histone cluster 1 H2A family member j	-1.313
17093724	ARHGEF39	Rho guanine nucleotide exchange factor 39	-1.294
16815090	CCNF	Cyclin F	-1.291
16868035	CTXN1	Cortexin 1	-1.271
16764791	KRT80	Keratin 80	-1.255
16822084	VPS9D1-AS1	VPS9D1 antisense RNA 1	-1.253
16922584	CHAF1B	Chromatin assembly factor 1 subunit B	-1.241
16989636	KIF20A	Kinesin family member 20A	-1.241
16869588	ASF1B	Anti-silencing function 1B histone chaperone	-1.230
16847432	BRIP1	BRCA1 interacting protein C-terminal helicase 1	-1.216
17074781	FAM86B2	Family with sequence similarity 86 member B2	-1.196
16714504	ZWINT	ZW10 interacting kinetochore protein	-1.194
17009482	CENPQ	Centromere protein Q	-1.190

Table S5. The top 30 most significant DEGs detected in hBMECs infected with *T. gondii* and treated with 0.1 μM monensin (TG_Monensin).

Gene Cluster ID	Gene Symbol	Gene Description	Log_2 FC
**16819325**	HERPUD1	Homocysteine inducible ER protein with ubiquitin-like domain 1	2.199
**16728284**	LOC105369371	Uncharacterized LOC105369371	5.135
**16979256**	SEC24D	SEC24 homolog D, COPII coat complex component	1.377
**16766578**	DDIT3	DNA damage inducible transcript 3	2.446
**16949292**	DNAJB11	DnaJ heat shock protein family (Hsp40) member B11	1.589
**16835125**	GOSR2	Golgi SNAP receptor complex member 2	1.894
**16975954**	SCFD2	Sec1 family domain containing 2	1.955
**16930066**	KDELR3	KDEL endoplasmic reticulum protein retention receptor 3	1.500
**16820076**	TMEM208	Transmembrane protein 208	1.486
**16837646**	TMEM104	Transmembrane protein 104	1.613
**16955442**	ARF4	ADP ribosylation factor 4	1.205
**16988703**	LMNB1	Lamin B1	-1.070
**16864181**	RCN3	Reticulocalbin 3	1.250
**17012079**	NUS1	NUS1 dehydrodolichyl diphosphate synthase subunit	1.266
**17090161**	GPR107	G protein-coupled receptor 107	1.548
**17066561**	POLR3D	RNA polymerase III subunit D	1.575
**16843511**	CCL5	C-C motif chemokine ligand 5	2.099
**16690511**	TAF13	TATA-box binding protein associated factor 13	2.186
**16957771**	TMEM39A	Transmembrane protein 39A	1.301
**17004903**	EDN1	Endothelin 1	-1.960
**16745186**	HYOU1	Hypoxia up-regulated 1	1.367
**16912057**	GZF1	GDNF inducible zinc finger protein 1	1.190
**17098411**	HSPA5	Heat shock protein family A (Hsp70) member 5	1.356
**16870047**	SLC35E1	Solute carrier family 35 member E1	1.324
**16941167**	MANF	Mesencephalic astrocyte derived neurotrophic factor	1.667
**16681827**	DHRS3	Dehydrogenase/reductase 3	-1.566
**16917054**	LRRN4	Leucine rich repeat neuronal 4	-1.390
**17007377**	SLC39A7	Solute carrier family 39 member 7	1.378
**17028426**	SLC39A7	Solute carrier family 39 member 7	1.378
**17031238**	SLC39A7	Solute carrier family 39 member 7	1.378

Table S6. The top 30 upregulated genes detected in 0.1 μM monensin treated and *T. gondii*-infected hBMECs (TG_Monensin) sorted with the highest $\log_2$ FC values at the top.

Gene Cluster ID	Gene Symbol	Gene Description	Log_2 FC
16728284	LOC105369371	Uncharacterized LOC105369371	5.135
16766578	DDIT3	DNA damage inducible transcript 3	2.446
16868827	YIPF2	Yip1 domain family member 2	2.229
16819325	HERPUD1	Homocysteine inducible ER protein with ubiquitin-like domain 1	2.199
16690511	TAF13	TATA-box binding protein associated factor 13	2.186
16843511	CCL5	C-C motif chemokine ligand 5	2.099
16941052	CYB561D2	Cytochrome b561 family member D2	2.008
17120058	PDE4DIP	Phosphodiesterase 4D interacting protein	1.989
16927633	SDF2L1	Stromal cell-derived factor 2 like 1	1.967
16975954	SCFD2	Sec1 family domain containing 2	1.955
16835125	GOSR2	Golgi SNAP receptor complex member 2	1.894
16774188	COG6	Component of oligomeric Golgi complex 6	1.860
16772733	GOLGA3	Golgin A3	1.822
16659133	NPPA-AS1	NPPA antisense RNA 1	1.763
16863287	FOSB	FosB proto-oncogene, AP-1 transcription factor subunit	1.756
16900053	EIF2AK3	Eukaryotic translation initiation factor 2 alpha kinase 3	1.744
16960844	VEPH1	Ventricular zone expressed PH domain containing 1	1.727
16775856	DNAJC3	DnaJ heat shock protein family (Hsp40) member C3	1.710
17103946	GPR173	G protein-coupled receptor 173	1.690
16941167	MANF	Mesencephalic astrocyte derived neurotrophic factor	1.667
16769481	ALDH1L2	Aldehyde dehydrogenase 1 family member L2	1.638
16934476	APOL2	Apolipoprotein L2	1.635
16848079	WIPI1	WD repeat domain, phosphoinositide interacting 1	1.628
16996176	MIR449A	MicroRNA 449a	1.625
16837646	TMEM104	Transmembrane protein 104	1.613
16861647	KCNK6	Potassium two-pore domain channel subfamily K member 6	1.607
16950686	SEC13	SEC13 homolog, nuclear pore and COPII coat complex component	1.602
16829139	MVD	Mevalonate diphosphate decarboxylase	1.600
16949292	DNAJB11	DnaJ heat shock protein family (Hsp40) member B11	1.589
16774112	UFM1	Ubiquitin-fold modifier 1	1.579

Table S7. The top 30 downregulated genes detected in 0.1 μM monensin treated and *T. gondii*-infected hBMECs (TG_Monensin) sorted with the lowest $\log_2$ FC values at the top.

Gene Cluster ID	Gene Symbol	Gene Description	$\log_2$ FC
17004903	EDN1	Endothelin 1	-1.960
16725041	FAM111B	Family with sequence similarity 111 member B	-1.929
17080595	DSCC1	DNA replication and sister chromatid cohesion 1	-1.752
16714747	RTKN2	Rhotekin 2	-1.664
16997676	MTRNR2L2	MT-RNR2 like 2	-1.642
17016400	HIST1H3F	Histone cluster 1 H3 family member f	-1.594
16800355	WDR76	WD repeat domain 76	-1.580
16986913	VCAN	Versican	-1.576
16681827	DHRS3	Dehydrogenase/reductase 3	-1.566
17122086	LINC01224	Long intergenic non-protein coding RNA 1224	-1.556
17016499	HIST1H1B	Histone cluster 1 H1 family member b	-1.546
16739636	SNHG1	Small nucleolar RNA host gene 1	-1.503
16716124	CERNA2	Competing endogenous lncRNA 2 for microRNA let-7b	-1.498
16877473	GEN1	GEN1, Holliday junction 5' flap endonuclease	-1.488
17016403	HIST1H3G	Histone cluster 1 H3 family member g	-1.463
16932821	YPEL1	Yippee-like 1	-1.456
16736638	E2F8	E2F transcription factor 8	-1.428
16798216	SNORD116-24	Small nucleolar RNA, C/D box 116-24	-1.417
16917054	LRRN4	Leucine rich repeat neuronal 4	-1.390
17079293	CCNE2	Cyclin E2	-1.379
17072622	LOC101927657	Uncharacterized LOC101927657	-1.371
16707695	HELLS	Helicase, lymphoid-specific	-1.365
17121990	ZNF730	Zinc finger protein 730	-1.357
16679411	EXO1	Exonuclease 1	-1.336
17016363	HIST1H3B	Histone cluster 1 H3 family member b	-1.321
16922584	CHAF1B	Chromatin assembly factor 1 subunit B	-1.321
17093724	ARHGEF39	Rho guanine nucleotide exchange factor 39	-1.317
17016366	HIST1H2AB	Histone cluster 1 H2A family member b	-1.305
16965346	NCAPG	Non-SMC condensin I complex subunit G	-1.298
16847432	BRIP1	BRCA1 interacting protein C-terminal helicase 1	-1.293

Table S8. The top 30 most significant DEGs induced in hBMECs 24 hours after *T. gondii* infection (TG_only).

Gene Cluster ID	Gene Symbol*	Gene Description	Log_2 FC
**16713762**	NA	NA	1.919
**16879863**	EPCAM	Epithelial cell adhesion molecule	2.249
**16811684**	CYP1A1	Cytochrome P450 family 1 subfamily A member 1	2.373
**17119058**	MIR4521	MicroRNA 4521	2.917
**16684080**	IFI6	Interferon alpha inducible protein 6	3.544
**16819224**	MT1M	Metallothionein 1M	4.414
**17009760**	FAM83B	Family with sequence similarity 83 member B	2.290
**17023646**	CTGF	Connective tissue growth factor	-1.650
**16707184**	IFIT3	Interferon-induced protein with tetratricopeptide repeats 3	2.167
**16857886**	ANGPTL4	Angiopoietin-like 4	3.262
**17009093**	VEGFA	Vascular endothelial growth factor A	1.800
**16666485**	IFI44L	Interferon induced protein 44 like	2.276
**16924592**	CYYR1	Cysteine and tyrosine-rich 1	1.685
**16830158**	WSCD1	WSC domain containing 1	1.877
**16820463**	CDH3	Cadherin 3	2.954
**16835158**	ITGB3	Integrin subunit beta 3	1.958
**17097661**	TNC	Tenascin C	1.833
**16697370**	PTGS2	Prostaglandin-endoperoxide synthase 2	2.512
**17023469**	NA	NA	3.432
**16723318**	PRRG4	Proline rich and Gla domain 4	2.008
**16705961**	DDIT4	DNA damage inducible transcript 4	2.453
**16702175**	PFKFB3	6-phosphofructo-2-kinase/fructose-2,6-biphosphatase 3	1.577
**16724633**	PTPRJ	Protein tyrosine phosphatase, receptor type J	2.022
**16800242**	NA	NA	1.643
**16914264**	WFDC2	WAP four-disulfide core domain 2	1.729
**16893349**	SNED1	Sushi, nidogen and EGF like domains 1	2.095
**16897834**	EFEMP1	EGF containing fibulin extracellular matrix protein 1	-1.688
**17105005**	NA	NA	1.352
**16672390**	IFI16	Interferon gamma inducible protein 16	1.370
**16931815**	SHANK3	SH3 and multiple ankyrin repeat domains 3	1.506

* NA denotes not available.

Table S9. The top 30 upregulated genes detected in *T. gondii*-infected hBMECs (TG_only) sorted with the highest $\log_2$ FC at the top.

Gene Cluster ID	Gene Symbol	Gene Description	Log_2 FC
16819224	MT1M	Metallothionein 1M	4.414
16684080	IFI6	Interferon alpha inducible protein 6	3.544
16717520	SNORA12	Small nucleolar RNA, H/ACA box 12	3.406
16857886	ANGPTL4	Angiopoietin-like 4	3.262
16728284	LOC105369371	Uncharacterized LOC105369371	3.066
16798132	SNORD116-1	Small nucleolar RNA, C/D box 116-1	3.010
16820463	CDH3	Cadherin 3	2.954
17119058	MIR4521	MicroRNA 4521	2.917
16834091	IGFBP4	Insulin-like growth factor binding protein 4	2.890
16843241	MYO1D	Myosin ID	2.775
16720085	IFITM1	Interferon induced transmembrane protein 1	2.724
16830837	SCARNA21	Small Cajal body-specific RNA 21	2.534
16850216	SECTM1	Secreted and transmembrane 1	2.524
16697370	PTGS2	Prostaglandin-endoperoxide synthase 2	2.512
16705961	DDIT4	DNA damage inducible transcript 4	2.453
17046586	SNORA22	Small nucleolar RNA, H/ACA box 22	2.383
16707196	IFIT1	Interferon-induced protein with tetratricopeptide repeats 1	2.379
16811684	CYP1A1	Cytochrome P450 family 1 subfamily A member 1	2.373
16962316	LIPH	Lipase H	2.347
17009760	FAM83B	Family with sequence similarity 83 member B	2.290
16815925	SNX29	Sorting nexin 29	2.285
16666485	IFI44L	Interferon induced protein 44 like	2.276
16879721	EPAS1	Endothelial PAS domain protein 1	2.276
16666509	IFI44	Interferon induced protein 44	2.274
16912362	ID1	Inhibitor of DNA binding 1, HLH protein	2.253
16879863	EPCAM	Epithelial cell adhesion molecule	2.249
16854301	ANKRD29	Ankyrin repeat domain 29	2.212
17059771	SAMD9	Sterile alpha motif domain containing 9	2.200
16968735	HERC6	HECT and RLD domain containing E3 ubiquitin protein ligase family member 6	2.182
17061099	RASA4	RAS p21 protein activator 4	2.181

Table S10. The top 30 downregulated genes detected in *T. gondii*-infected hBMECs (TG_only) sorted with the lowest $\log_2$ FC at the top.

Gene Cluster ID	Gene Symbol	Gene Description	Log_2 FC
**17062941**	MIR29A	MicroRNA 29a	-3.201
**16702257**	LINC00707	Long intergenic non-protein coding RNA 707	-2.370
**16686273**	RNU5D-1	RNA, U5D small nuclear 1	-2.161
**16686271**	RNU5F-1	RNA, U5F small nuclear 1	-2.050
**16844585**	KRTAP2-3	Keratin associated protein 2-3	-1.971
**16708260**	OLMALINC	Oligodendrocyte maturation-associated long intergenic Non-coding RNA	-1.856
**17117888**	ZEB2	Zinc finger E-box binding homeobox 2	-1.850
**16745503**	BLID	BH3-like motif containing, cell death inducer	-1.830
**16683268**	LINC01355	Long intergenic non-protein coding RNA 1355	-1.734
**16970188**	LOC645513	Septin 7 pseudogene	-1.706
**16809748**	MNS1	Meiosis specific nuclear structural 1	-1.694
**16897834**	EFEMP1	EGF containing fibulin extracellular matrix protein 1	-1.688
**16909524**	SNORD82	Small nucleolar RNA, C/D box 82	-1.682
**16677898**	LOC100287497	Septin 7 pseudogene	-1.672
**17122654**	CYTOR	Cytoskeleton regulator RNA	-1.666
**17023646**	CTGF	Connective tissue growth factor	-1.650
**16900293**	ANKRD36C	Ankyrin repeat domain 36C	-1.649
**16745507**	MIRLET7A2	MicroRNA let-7a-2	-1.626
**17020480**	GUSBP4	Glucuronidase, beta pseudogene 4	-1.616
**16697660**	MIR181B1	MicroRNA 181b-1	-1.578
**16900286**	LINC00342	Long intergenic non-protein coding RNA 342	-1.545
**17051553**	CPA4	Carboxypeptidase A4	-1.537
**17124806**	LINC-PINT	Long intergenic non-protein coding RNA, p53 induced transcript	-1.536
**17109714**	SCARNA9L	Small Cajal body-specific RNA 9-like	-1.507
**16836624**	MIR21	MicroRNA 21	-1.476
**17062945**	LINC-PINT	Long intergenic non-protein coding RNA, p53 induced transcript	-1.467
**17122664**	CYTOR	Cytoskeleton regulator RNA	-1.462
**17087768**	NIPSNAP3B	Nipsnap homolog 3B	-1.422
**17124904**	LINC-PINT	Long intergenic non-protein coding RNA, p53 induced transcript	-1.407
**17072622**	LOC101927657	Uncharacterized LOC101927657	-1.406
